# Path planning for volumetric flask grasping based on visual guidance and multi-constraint optimization

**DOI:** 10.1371/journal.pone.0347043

**Published:** 2026-04-20

**Authors:** Zhaopeng Yuan, Meng Li, Chengyun Wang

**Affiliations:** School of Electrical Engineering, University of Jinan, Jinan, China; Southeast University, CHINA

## Abstract

In the automated operation of the chemical laboratory, using a robotic arm to pick up volumetric flasks is a core step of the operation process. By implementing reasonable path planning, the grasping operation of the robotic arm can be made efficient and precise. In this scenario, the traditional Rapidly-exploring Random Tree Star (RRT*) algorithm suffers from low sampling efficiency and numerous sharp path turns. To address these problems, this paper proposes the Vision-guided Multi-constraint RRT* (VM-RRT*) algorithm, which integrates visual guidance sampling and multi-constraint paths. Firstly, the algorithm determines the spatial coordinates of the volumetric flask through target detection, reducing ineffective exploration and accelerating path convergence. Subsequently, it uses cubic B-splines to fit the path and improves the density of data points through spline interpolation methods. Combined with low-pass filtering, it further reduces noise and eliminates sudden changes in the end-effector speed and acceleration of the robotic arm, realizing multi-constraint trajectory optimization. Experimental simulation results show that the average planning time of the VM-RRT* algorithm is 3.27 seconds, which is approximately 20% shorter than that of the traditional RRT* algorithm (4.07 seconds), effectively improving the experimental efficiency. At the same time, the end motion parameters of the robotic arm are effectively controlled, providing support for laboratory automation.

## 1. Introduction

With the continuous advancement of laboratory automation technology, robotic arms have been widely adopted in precision operations across fields such as chemistry and biology [1]. Their manipulation of volumetric flasks and pipetting processes constitutes a critical step in precise experimental workflows [2]. Due to the confined space and numerous obstacles (lab benches, reagent racks, other glassware, etc.) in laboratory environments, coupled with the high precision demands of this task, robotic arm movement paths must avoid collisions with any obstacles. Additionally, the end-effector must move smoothly to minimize liquid agitation while also responding rapidly to enhance efficiency. Consequently, path planning for robotic arms during this process is particularly critical.

Traditional path planning algorithms like the Rapidly-exploring Random Tree (RRT) [3–5] and RRT* [6,7] have been applied in path search due to their probabilistic completeness, yet they exhibit limitations. On one hand, random sampling often places most points in ineffective regions like beneath the workbench, slowing convergence toward the flask target position. On the other hand, the generated paths frequently contain sharp-angled turns, causing abrupt changes in end-effector angular acceleration and significant vibration at the end effector. This increases the risk of liquid spillage from the flask, rendering the approach unsuitable for precision operations.

Relevant domestic and international studies have addressed the shortcomings of traditional RRT algorithms in robotic arm path planning, such as poor adaptability in high-dimensional spaces, excessive sampling blindness, and low convergence efficiency. Multiple improvement schemes have been proposed, primarily focusing on algorithmic performance optimization. For high-dimensional obstacle avoidance in robotic arms, Yu, Q. et al. [8] proposed an improved RRT algorithm that employs Monte Carlo methods to evaluate the robot’s reachable workspace, thereby defining motion boundaries. It incorporates collision feedback to guide adaptive tree expansion and adjust sampling directions, alongside a global adaptive step size strategy for rapid initial path generation. After optimization, this method effectively reduces redundant sampling while enhancing trajectory smoothness. MATLAB-based 2D/3D multi-environment simulations and physical robotic arm experiments demonstrated significant advantages over traditional algorithms like standard RRT, Bidirectional Rapidly-exploring Random Tree (Bi-RRT), and G-RRT in average runtime, iteration count, and path cost. To address RRT’s issues of poor node validity and slow convergence, Gu, Y. et al. [9] proposed the LRRT* algorithm — a two-stage planning approach that employs a goal-directed strategy during initial path generation to guide node expansion toward target points, while re-generating nodes that fail collision detection using a Levy flight strategy to enhance expansion point quality. The optimization stage identifies effective sampling regions through map partitioning and introduces a node rejection strategy to eliminate high-cost nodes. In 3D environments, LRRT* reduces initial path planning time by 91.9% compared to RRT* in 3D environments and stably generates collision-free trajectories when validated on the UR5 robotic arm. To resolve the issues of scattered sampling points, slow expansion due to fixed stride length, and excessive path turns in the RRT-Connect algorithm, Wang, Z. et al. [10] proposed the DAPF-RRT algorithm; this approach integrates a target-bias strategy to reduce sampling blindness, a dynamic step-size mechanism to adjust expansion efficiency based on obstacle distance, and an artificial potential field function to constrain node growth direction. Post-planning, through greedy pruning and cubic B-spline smoothing, path length was reduced by 15.4% and planning time decreased by 49.2% compared to RRT-Connect, adapting to robotic arm motion characteristics.

Beyond robotic arm scenarios, researchers have tailored RRT improvements for path planning in mobile robots, UAVs, and other devices. For balanced global exploration and convergence efficiency, S. Ganesan et al. [11] proposed the RRT*-NUS algorithm* — it integrates non-uniform and uniform hybrid sampling strategies, reducing planning time by 67.5% compared to traditional RRT, achieving a threefold improvement in convergence rate, and outperforming baseline algorithms across multiple performance metrics. For redundant robotic arm obstacle avoidance, J. Dai et al. [12] proposed the Potential-Guided Bidirectional RRT*, which integrates artificial potential fields with direct-connection strategies in joint space to yield shorter paths while circumventing complex inverse kinematics calculations; For narrow-channel scenarios, Uwacu D et al. [13] proposed the HAS-RRT algorithm that employs a workspace topology-guided approach, prioritising exploration of skeleton-indicated paths through hierarchical planning strategies and maintaining robustness when guidance information fails; For path cost reduction, X. Cui et al. [14] proposed the MQ-RRT*, which reduces path cost through sparse sampling and dynamic target bias strategies, outperforming algorithms like RRT* and Q-RRT. For UAV path planning, Wilson TS et al. [15] proposed the AORRTC algorithm that extends RRT-Connect into an asymptotically optimal planner; for high-dimensional complex planning problems involving 7–8 degree-of-freedom robots, it achieves faster solution speeds and greater stability compared to comparable optimal algorithms. For disinfection robots, H. Wang et al. [16] proposed the APF-GFARRT* algorithm that integrates artificial potential fields with fuzzy adaptive stride, reducing initial path search time by 46.52% and lowering path cost by 10.01%. To enhance node utilization and convergence rate, J. Ding et al. [17] proposed the EP-RRT* algorithm that generates heuristic sampling regions by integrating the RRT-Connect* greedy strategy. To improve path quality and iteration count, H. Wang et al. [18] proposed the IBPF-RRT*, which outperforms five comparative algorithms through edge potential fields and bidirectional pruning. To avoid congested areas while optimizing convergence speed and path cost, Y.-m. Liang et al. [19] proposed the CCPF-RRT* (incorporating a mobility cost function) and J. Wang et al. [20] proposed the FCF-RRT* (employing a path cost function).

Existing improved algorithms combine techniques like target bias, bidirectional expansion, and adaptive stride, or employ methods such as B-splines and artificial potential fields, to enhance search efficiency or path smoothness, addressing shortcomings of traditional path planning algorithms. However, these approaches struggle to simultaneously meet the demands of rapid convergence, path smoothness, and high-precision focusing on the bottle area in bottle-grabbing scenarios. Particularly in confined spaces and complex obstacle scenarios, they cannot balance convergence speed and path smoothness, failing to ensure efficiency and safety during flask grasping. To address these issues, this paper improves the RRT* algorithm by proposing the VM-RRT* framework. Visual guidance sampling focuses on the flask’s vicinity, enabling the algorithm to concentrate on the flask area, reduce redundant exploration, and accelerate convergence. Simultaneously, by integrating cubic B-spline curve fitting, third-order Butterworth low-pass filtering, and joint kinematic constraints, it achieves dual smoothing of path geometry and kinematics. This ensures smooth robotic arm motion during laboratory volumetric flask grasping, preventing liquid agitation within the flask.

## 2. Problem statements

### 2.1. Research scenario and core issues

This study focuses on the flask-grasping task within automated operations in chemical laboratories. The operational environment exhibits the following characteristic features:

(1) Laboratory interiors feature confined spaces with densely distributed obstacles such as laboratory benches, reagent racks, and glassware, severely restricting the robotic arm’s manoeuvring space and necessitating collision avoidance in path planning;
(2) Volumetric flasks contain chemical reagents; vibrations or abrupt movements from the robotic arm’s end-effector may cause liquid agitation and spillage, necessitating smooth trajectories with stable velocity and acceleration;
(3) As the primary operational target, volumetric flasks require rapid spatial localisation and path guidance towards the target area, avoiding inefficient exploration caused by traditional random sampling.

Based on these scenario characteristics, the core challenges in current robotic arm path planning can be summarised as follows:

Traditional path planning algorithms (e.g., RRT, RRT*) employ globally uniform random sampling, resulting in slow convergence;

Paths generated by planning algorithms often exhibit polygonal characteristics with numerous sharp-angled junctions, causing abrupt angular acceleration changes at the robotic arm’s end-effector and inducing end-effector vibration;

In scenarios with dense obstacles or partially obscured targets, existing algorithms struggle to balance rapid convergence with obstacle avoidance, frequently resulting in path redundancy or collision risks.

### 2.2. Fundamental algorithms (RRT and RRT*) principles

**Fig 1:**
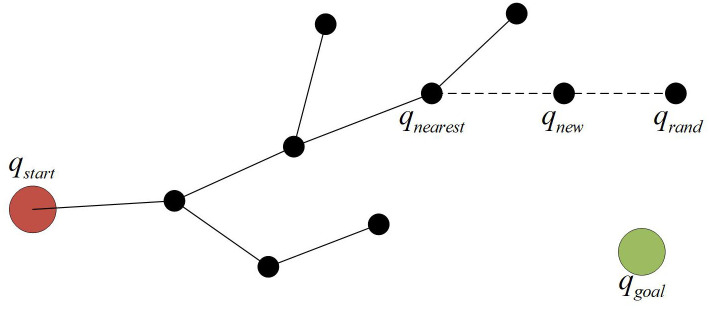
Core process diagram of the RRT algorithm.

To solve the above path planning problem, it is necessary to improve the classical sampling-based algorithms. The following briefly introduces the core logic of the RRT and RRT* algorithms that the algorithm design in this paper relies on:

RRT (Rapidly-exploring Random Tree) is a classic algorithm for path planning in multi-dimensional space, and it is probabilistically complete. The basic process is shown in Fig 1 below.

Initialization: Take the initial position *q*_start_ as the root node and construct an empty path tree;

Random sampling: Generate a sampling *q*_rand_ randomly in the motion space;

Node search: In the constructed path tree, find the node *q*_rand_ that is closest to *q*_nearest_ and has no obstacles blocking the connection;

Node expansion: Based on the relative position of *q*_rand_ and *q*_nearest_, generate a new node *q*_new_ with a set step size and add *q*_new_ to the path tree;

Termination judgment: Repeat the above steps until the target point is included in the path tree and the path planning is completed.

However, this algorithm has obvious limitations in the laboratory environment: the random sampling is not goal-oriented, the utilization rate of sampling points is low, and the generated path is a discrete polyline, which cannot meet the smoothness requirements for the volumetric flask grasping.

RRT* is a deep optimization of the traditional RRT algorithm. Its main idea lies in progressive optimization of the path. On the basis of retaining the sampling, connection, and expansion of the conventional process, two additional steps of reselecting the parent node and re-routing are added.

**Fig 2:**
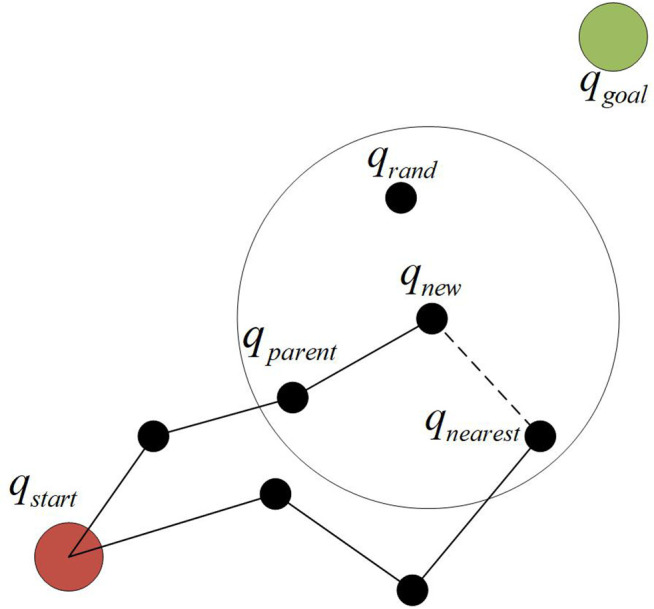
Step of re-selecting parent node.

In the reselecting parent node step, a neighborhood centered on *q*_new_ is selected, and the path tree nodes that already exist in the neighborhood (such as *q*_nearest_, *q*_parents_, etc.) are traversed. The original parent node of *q*_new_ was *q*_nearest_. If it is found that *q*_parents_ can optimize the path cost from *q*_new_ to the root node *q*_start_, then *q*_parent_ is selected as the parent node of *q*_new_, as shown in Fig 2. By locally replacing the parent node of the node with a node within the neighborhood, the path cost of *q*_new_ (i.e., the path cost to the starting point) is reduced (i.e., the path cost is optimized preliminarily).

In this study, the parent node selection and subsequent rewiring phases employ a unified path cost function, defined as the sum of the Euclidean distances between discrete nodes in the configuration space. The mathematical expression is:


$$J(q)=\sum_{i=1}^{n}\|q_{i}-q_{i-1}{\|}_{2}$$ (1)

Where, *q*_0_ = *q*_start_ is the starting node of the planning process, *q*_*n*_ is the current node to be optimized, and *J*(*q*) represents the path cost. $\|\cdot {\|}_{2}$ denotes the L2 norm, which is used to calculate the Euclidean distance between two nodes in the configuration space.

**Fig 3:**
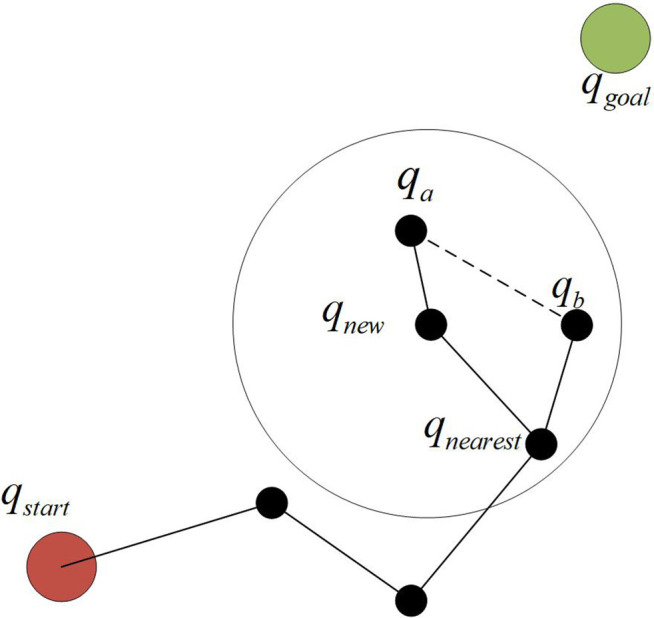
Re-wiring phase.

In the re-wiring process, the logic was further optimized. Starting from *q*_new_, the existing path tree nodes in the neighborhood were traversed. The original parent node of *q*_a_ was *q*_b_. If *q*_new_ was set as its own parent node and the path cost to *q*_start_ was reduced by doing so, then this node would be connected to the path tree through *q*_new_, as shown in Fig 3.

By optimizing the connection paths of the local nodes in this way, the overall path cost is reduced. The paths are obtained through continuous iterative sampling of new nodes, then the local paths are optimized through the re-selection of parent nodes, the re-routing process further optimizes the neighborhood connections, and finally, when *q*_goal_ is introduced into the path tree, the path planning route is completed.

### 2.3. Spatial definition and planning process description

To clarify the mathematical spatial foundation for algorithm execution and prevent spatial mapping ambiguities during path planning, the following spatial definitions, core functions, and overall planning specifications are hereby provided:

The *workspace* (𝒲_space_) is the three-dimensional physical space ($𝒲\in {\mathbb{R}}^{3}$) accessible by the robotic arm’s end-effector. The target coordinates *P*_goal_ of the flask are located within this space, whose boundaries are defined by the physical environment of the laboratory. The *configuration space* (𝒞_space_) corresponds to the joint angle combination space of the 6-degree-of-freedom AUBO I5 robotic arm, whose elements are the joint angle vector $q=[q_{1};q_{2};\ldots ;q_{6}{]}^{T}$ (each joint angle conforms to hardware limit requirements). For the 6-degree-of-freedom pure rotational joint AUBO I5 robotic arm (without mobile or flexible components), the configuration space is equivalent to the joint space. The core of path planning involves searching for collision-free paths in 𝒞_space_. Specifically, the *free space* (*C*_free_) represents the set of joint configurations (i.e., ${𝒞}_{\text{free}}=\{q\in 𝒞|\text{CollisionCheck}(q)=\text{False}\}$) that avoid collisions, while the obstacle space (*C*_obs_) constitutes its complement—a region the algorithm must completely avoid.

All algorithmic functions are computed in 𝒞space. Although the sampling process in this paper generates target biased sampling points *P*_sample_ in 𝒲_space_, these must be mapped to joint configurations *q*_sample_ in 𝒞space via inverse kinematics (Newton-Raphson method). CollisionCheck(*q*_sample_) then verifies whether *q*_sample_ belongs to the ${𝒞}_{\text{free}}$ category. If mapping fails (no feasible joint configuration exists) or collision occurs, resampling is performed. To address the multi-solution and singularity issues in inverse kinematics, this paper employs corresponding handling strategies: When solving inverse kinematics for *P*_sample_, if multiple feasible joint configurations exist, the one closest to the current path tree node *q*_nearest_ is selected to minimize joint displacement. When detecting a joint configuration approaching a singular configuration (where the determinant of the Jacobian matrix approaches zero), the inverse kinematics is re-solved by slightly adjusting the sampling point *P*_sample_ to prevent the robotic arm from entering a motion singular state.

The overall planning process is as follows: First, determine the target point in 𝒲_space_. Then, map it to the joint configuration in 𝒞space using the Newton-Raphson method. Next, perform path search within the ${𝒞}_{\text{free}}$ in 𝒞space. Finally, further ensure motion stability through joint velocity constraints, ultimately outputting a collision-free, smooth path.

## 3. Algorithm design and analysis

### 3.1. Design of a vision-guided hybrid sampling mechanism

The RRT^*^ algorithm uses uniform random sampling, which results in a slow convergence speed in the target area and is difficult to meet the requirements for efficient and high-precision operations. Therefore, this paper proposes a visual-guided sampling target orientation mechanism that deeply integrates machine vision and path planning logic, and constructs a sampling target orientation.

This mechanism, based on the YOLOv11 target detection model, corely converts visual information into the robotic arm’s three-dimensional coordinates (denoted as *P*_goal_). During model inference, in addition to outputting the three-dimensional coordinates of the volumetric flask, the model also outputs a trust degree indicator *c* ranging from 0 to 1, with a larger value indicating higher reliability.

However, target detection results are not fully reliable due to lighting, occlusion and other factors. A single sampling method may lead to rapid convergence to the target area when detection is accurate, but it may converge to an invalid area when detection is erroneous.

To balance the convergence efficiency in the target region and the algorithm’s probabilistic completeness, this paper proposes a hybrid sampling model, as formulated in (2).


$$P_{\text{sample}}=\alpha \cdot P_{\text{Gaussian}}+(1-\alpha )\cdot P_{\text{Uniform}}$$ (2)

where *P*_Gaussian_ denotes target-biased Gaussian sampling and *P*_Uniform_ represents global uniform sampling in 𝒞_space_. By retaining a proportion of global uniform samples, the model ensures a non-zero sampling density within the free space ${𝒞}_{\text{free}}$, thereby preserving the algorithm’s core property of probabilistic completeness.

#### 3.1.1. Probability completeness verification and dynamic sampling strategies for hybrid sampling model.

Probability completeness is a core property of path planning algorithms, whose fundamental requirement is that if a feasible path exists between the start and goal points, the algorithm will inevitably find a valid path provided the planning time is sufficiently long. This property presupposes that the sampling distribution satisfies a non-zero probability density everywhere on the obstacle-free configuration space *C*_free_.

In the hybrid sampling model proposed herein, even under high-confidence scenarios, the globally uniform sampling component retains at least 20% coverage (α≤0.8) of the workspace. This component can cover the entire workspace 𝒲. Simultaneously, the support set of the Gaussian distribution component $𝒩(P_{\text{goal}},\sigma^{2}I)$ spans the entire space (with no regions of zero probability). Given that 𝒩(0,I) is a continuous distribution with support set ${\mathbb{R}}^{n}$, combined with the non-zero proportion of global uniform sampling, the probability density function of the hybrid distribution satisfies *p*(*q*)>0 at any point within 𝒞_free_. This fulfils the necessary and sufficient condition for probabilistic completeness. Consequently, the probability density of the hybrid distribution within *C*_free_ remains perpetually greater than zero, fulfilling the core prerequisite for probabilistic completeness. Compared to pure Gaussian biased sampling, this model retains the enhancement of planning efficiency through visual bias while ensuring probabilistic completeness.

At the same time, during path planning, the algorithm performs inverse kinematics calculations on the sampling points and evaluates the mapping from the workspace to the joint space, thereby eliminating invalid nodes that cannot yield valid joint angles or violate motion constraints. This pruning process excludes only infeasible configurations and does not affect the algorithm’s search coverage within the feasible configuration space; therefore, even when accounting for inverse kinematics constraints and spatial mapping, the algorithm still satisfies probabilistic completeness.

Based on the dynamic variation of visual detection confidence, this paper designs two distinct sampling strategies. The logical relationship between core parameters and functions is as follows: α controls the probability proportion of Gaussian sampling, the Gaussian sampling expression defines the generation rules for sampling points, and the noise coefficient determines the dispersion of sampling points.

**Fig 4:**
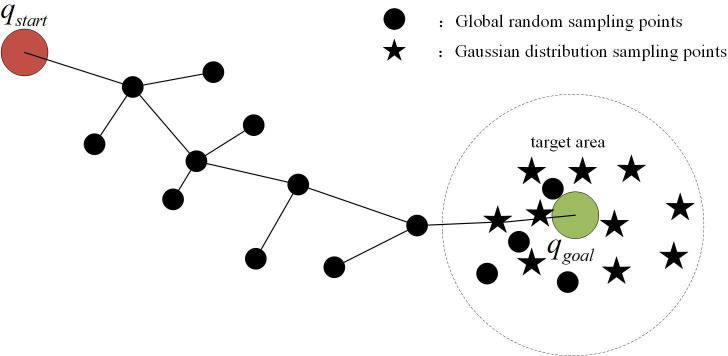
Schematic diagram of high confidence sampling strategy.

When *c* > 0.7, this is designated as a high-confidence scenario. At this point, the reliability of detection results is strong, and Gaussian distribution sampling points should be concentrated as much as possible near the target area to facilitate rapid path convergence, as shown in Fig 4. The hybrid sampling weight α is then set to [0.7, 0.8], with the corresponding mathematical expression for Gaussian bias sampling being:


$$P_{\text{Gaussian}}=P_{\text{goal}}+0.2\times \text{size}(𝒲)\times 𝒩(0,I)$$ (3)

Among these, 𝒩(0,I) denotes multivariate Gaussian noise, whose probability distribution follows a normal distribution centred on the mean, ensuring sampling points are uniformly distributed around the target. The noise coefficient of 0.2 represents the optimal value determined through multi-confidence cross-experiments as detailed in Section 3.2. Parameter α governs the execution probability of this Gaussian sampling rule, while the noise coefficient controls the dispersion of sampling points around the target region.

**Fig 5:**
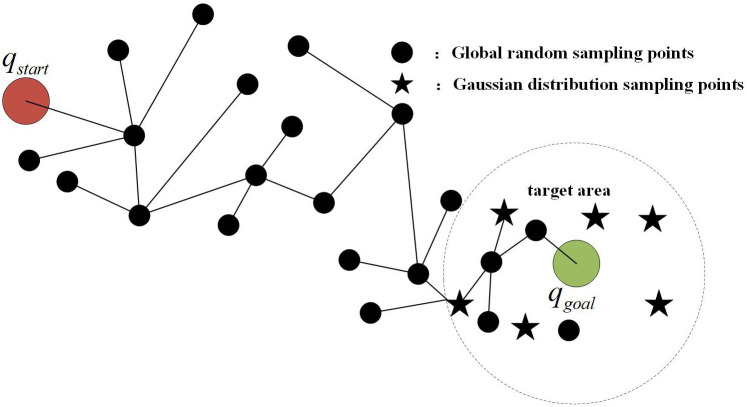
Schematic diagram of low confidence sampling strategy.

When *c* ≤ 0.7, detection confidence diminishes, rendering results susceptible to interference and bias. To prevent sampling from becoming trapped in invalid regions, enhancing global exploration capability is necessary. At this point, the hybrid sampling weight α is adjusted to [0.3, 0.6], with the corresponding Gaussian sampling expression similarly presented in (3). Under this configuration, the reduced value of α directs sampling points towards a more global distribution. Meanwhile, the noise coefficient of 0.2 induces a slight tendency for Gaussian sampling points to concentrate within the target region. This ensures sampling points extend into surrounding areas without entirely departing from the target orientation, as shown in Fig 5.

The core of the aforementioned strategy lies in dynamically adjusting sampling points around target locations. However, the detection results output by the YOLOv11 model are by default based on the image coordinate system, rendering them directly unusable for robotic arm recognition and utilisation. Consequently, camera calibration parameters must be determined, and coordinate transformation formulas applied to map detection results from the image coordinate system to the robotic arm’s base coordinate system.

First, camera calibration is employed to obtain internal and external parameters, completing image distortion correction and the transformation from the image coordinate system to the camera coordinate system. Subsequently, methods such as hand-eye calibration are used to derive the transformation matrix from the camera coordinate system to the base coordinate system, incorporating both rotational and translational information. Finally, this matrix is substituted into the following coordinate transformation formula (4):


$$P_{\text{base}}=T_{\text{cam2base}}\cdot P_{\text{image}}$$ (4)

Here, *P*_image_ denotes the point coordinates within the image coordinate system, while *T*_cam2base_ represents the transformation matrix from the camera coordinate system to the base coordinate system. This matrix converts detection points from the image coordinate system to *P*_base_ within the base coordinate system, thereby defining the spatial position of the volumetric flask and establishing the coordinate foundation for efficient execution of the sampling strategy.

#### 3.1.2. Algorithmic implementation process of sampling strategies.

For the VM-RRT^*^ main algorithm, the target pose *P*_goal_ in the workspace is first mapped to the joint configuration *q*_goal_ in the configuration space via inverse kinematics, while a path tree rooted at the starting joint configuration *q*_init_ is initialised. In each iteration, the SampleFree function is invoked to sample the joint configuration *q*_sample_ in the configuration space, expanding new nodes and filtering collision-free nodes through collision detection. Subsequent steps optimise the path cost by reconnecting with neighbouring nodes. After extracting the original path, it undergoes B-spline smoothing and post-smoothing collision detection, finally outputting a safe, feasible, and smooth trajectory.

Key parameters involved in the subsequent algorithm iteration are clearly defined as follows: the rewiring neighborhood radius *r*_rewire_ is set to 550 mm, which matches the sampling step size to balance path optimization efficiency and computational cost; the maximum number of iterations for inverse kinematics solving *N*_ik_ is 100 to ensure the solving process converges within a reasonable time frame; the size(𝒲) refers to the three-dimensional size vector of the workspace 𝒲, calculated as $\left[X_{\text{max}}-X_{\text{min}},Y_{\text{max}}-Y_{\text{min}},Z_{\text{max}}-Z_{\text{min}}\right]$, which is derived from the AUBO I5 robotic arm hardware parameters and consistent with the workspace definition in Section 2.3.

Algorithm 1. VM-RRT* Main Algorithm

**Require:** Workspace 𝒲 (joint space/end-effector workspace bounds),

*q*_init_ (start joint configuration), *P*_goal_ (target goal pose),

*K* (robotic arm kinematic constraints), *O* (obstacle set in workspace),

*N*_max_ (maximum iteration number), c(·) (visual confidence score function),

Δq (joint step size for steering, set to 50 mm)

**Ensure:** Path (collision-free and smoothed path from *q*_init_ to *q*_goal_)

1: Initialize tree *T*, set $T.\text{root}\leftarrow q_{\text{init}}$ and $T.\text{cost}(q_{\text{init}})\leftarrow 0$

2: $q_{\text{goal}}\leftarrow \text{InverseKinematics}(P_{\text{goal}},K)$

3: **for** *i* = 1 **to** *N*_max_ **do**

4:  $P_{\text{sample}}\leftarrow \text{SampleFree}(c,𝒲,P_{\text{goal}})$

5:  $q_{\text{sample}}\leftarrow \text{InverseKinematics}(P_{\text{sample}},K)$

6:  $q_{\text{nearest}}\leftarrow \text{NearestNode}(T,q_{\text{sample}})$

7:  $q_{\text{new}}\leftarrow \text{Steer}(q_{\text{nearest}},q_{\text{sample}},\Delta q)$

8:  **if** $\neg \text{CollisionCheck}(q_{\text{new}},O,K)$ **then**

9:   $\text{AddNode}(T,q_{\text{new}},q_{\text{nearest}})$

10:   $q_{\text{near\_set}}\leftarrow \text{NeighborNodes}(T,q_{\text{new}},r_{\text{rewire}})$

11:   $q_{\text{parent\_opt}}\leftarrow \text{SelectOptimalParent}(T,q_{\text{new}},q_{\text{near\_set}})$

12:   $\text{UpdateNodeParent}(T,q_{\text{new}},q_{\text{parent\_opt}})$

13:   $\text{RewireTree}(T,q_{\text{new}},q_{\text{near\_set}})$

14:  **end if**

15: **end for**

16: $\text{RawPath}\leftarrow \text{ExtractPath}(T,q_{\text{init}},q_{\text{goal}})$

17: Path←SmoothPath(RawPath)

18: Path←PostSmoothingCollisionCheck(Path,O)

19: **return** Path

The SampleFree function sets explicit thresholds based on visual detection confidence *c* and adjusts the sampling strategy accordingly. At high confidence levels (*c* > 0.7), it employs Gaussian biased sampling with a weight of 0.8, concentrating on target regions to accelerate algorithm convergence. At low confidence levels (*c* ≤ 0.7), the Gaussian sampling weight is reduced to 0.4, combined with uniform sampling across the entire workspace to ensure global exploration capability. Furthermore, the function incorporates a sampling point clipping step, which guarantees that the output sampling points remain strictly within the workspace bounds and prevents invalid kinematic mapping. The SmoothPath function implements cubic B-spline curve fitting and third-order Butterworth low-pass filtering, while the PostSmoothingCollisionCheck function adopts discrete point verification with 5 mm interval and continuous segment validation to ensure collision safety.

**Algorithm 2. SampleFree Adaptive Sampling Function**

**Require:** *c* (visual confidence score, range [0,1]),

𝒲 (workspace bounds), *P*_goal_ (target goal pose)

**Ensure:** *P*_sample_ (valid sample pose within workspace 𝒲)

1: Set Gaussian sampling weight α based on confidence *c*

2: **if** *c* > 0.7 **then**

3:   α←0.8

4: **else**

5:   α←0.4

6: **end if**

7: $P_{\text{Gaussian}}\leftarrow P_{\text{goal}}+0.2\times \text{size}(𝒲)\times 𝒩(0,I)$

8: $P_{\text{Uniform}}\leftarrow \text{UniformSample}(𝒲)$

9:  $P_{\text{sample}}\leftarrow \alpha \cdot P_{\text{Gaussian}}+(1-\alpha )\cdot P_{\text{Uniform}}$

10: $P_{\text{sample}}\leftarrow \text{ClipToWorkspace}(P_{\text{sample}},𝒲)$

11: **return** *P*_sample_

### 3.2. Multi-constraint trajectory smoothing optimization design

The gripping of volumetric flasks demands exceptionally smooth trajectories, as abrupt movements may cause liquid agitation or even spillage within the flask. Multi-constraint path optimization addresses both geometric and kinematic aspects of the path. This ensures collision-free operation while achieving smooth end-effector motion.

#### 3.2.1. Geometric smoothing of cubic B-spline curves.

**Fig 6:**
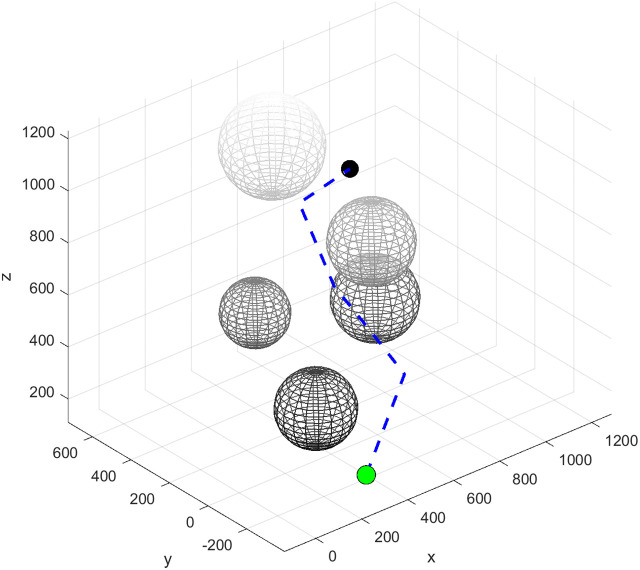
The path generated by the RRT* algorithm.

The geometric smoothing processing of the cubic B-spline curve is applied to the original path, which is obtained by connecting a large number of discrete points. When the robotic arm moves, the path will form multiple turning points, as shown in Fig 6.

Too many turning points will cause the movement direction of the robotic arm’s connecting rods to undergo sudden changes, increasing the possibility of collision with surrounding obstacles. Therefore, this paper adopts cubic B-spline curve fitting to smooth the path. The main idea is to use a set of ordered control points to determine a continuous differentiable spatial curve, ensuring that the position, direction and curvature of the robotic arm’s movement are strictly maintained as continuous. The mathematical expression of the cubic B-spline curve is given by (5):


$$P(u)=\sum_{i=0}^{n}D_{i}\cdot N_{i,3}(u)\;(u\in [0,1])$$ (5)

In the formula, *D*_*i*_ = [*x*_*i*_, *y*_*i*_, *z*_*i*_]^*T*^ represents the coordinate of the control point in the three-dimensional space, *N*_*i*,3_(*u*) is the cubic B-spline basis function whose shape is determined by the node vector $U=[u_{0},u_{1},...,u_{m}]$, where *m* = *n* + 4 and *n* represent the number of control points.

From the perspective of differential geometry, the smoothness of a curve can be quantitatively described by curvature, and the formula (6) is as follows:


$$\kappa (u)=\frac{|P^{\prime }(u)\times P^{\prime \prime }(u)|}{|P^{\prime }(u){|}^{3}}$$ (6)

Here, $P^{\prime }(u)=\frac{dP}{du}$ and $P^{\prime \prime }(u)=\frac{d^{2}P}{du^{2}}$ represent the first-order and second-order derivatives of the curve with respect to the parameter *u*. The first-order derivative indicates the direction of the tangent line at a certain point on the curve, while the second-order derivative represents the acceleration of curvature at that point. At the inflection points of the polyline path, the first-order derivative undergoes a sudden change, and the modulus of the second-order derivative increases, causing a jump in the curvature κ(u). This jump is transmitted to the end of the robotic arm through the joints and results in jittering, which affects the stability of the liquid in the volumetric flask.

**Fig 7:**
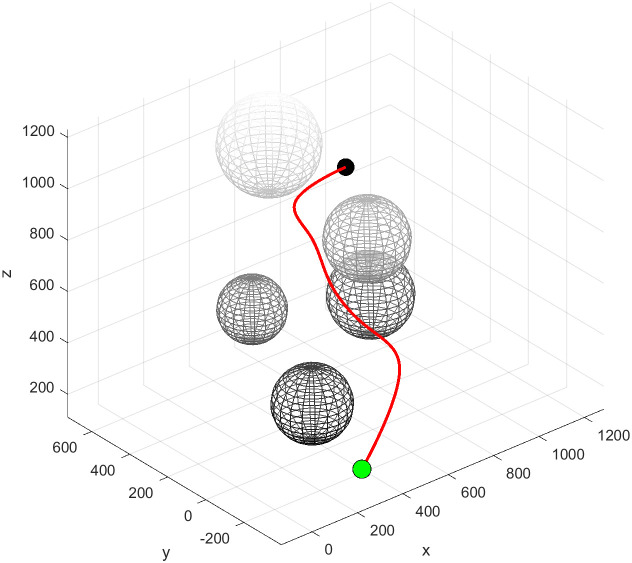
Path after cubic B-spline curve optimization.

The cubic B-spline basis functions exhibit continuity in both first and second derivatives. Through judicious selection of control points, global curvature smoothness is achieved, eliminating the possibility of curvature discontinuities. Following global fitting, the path undergoes a transformation from a polygon to a smooth curve, as illustrated in Fig 7. This establishes the geometric foundation for the safety and stability of flask gripping operations.

#### 3.2.2. Collision detection and correction process for smooth paths.

To mitigate trajectory corner drift collisions caused by B-spline fitting, this study incorporates a post-processing collision detection workflow after smoothing. First, discrete point verification is performed. The smoothed path is segmented into discrete nodes at 5 mm intervals. For each node, the *CollisionCheck* function is invoked on the corresponding joint configuration. The spatial pose of the robotic arm’s linkages is calculated via forward kinematics, then rapidly compared against the axis-aligned bounding box (AABB) of obstacles to perform overlap checks. This eliminates collision risks at individual points.

After completing discrete point verification, perform continuous segment validation on collision-free path segments: First, construct AABB bounding boxes for both the path segment and the obstacles. If there is no overlap in the projection intervals along any of the x, y, or z coordinate axes, the path segment is deemed collision-free. If the bounding boxes overlap, form a line segment using the path segment’s endpoints *P*_1_ and *P*_2_. Define vectors $\vec{P_{1}P_{2}}=P_{2}-P_{1}$ and $\vec{P_{1}O}=O-P_{1}$ based on the sphere’s center *O* and radius *r*. Calculate the line segment parameter $t=\frac{\vec{P_{1}O}\cdot \vec{P_{1}P_{2}}}{{\left\|\vec{P_{1}O}\right\|}^{2}}$ (constrained by t∈[0,1]). If the shortest distance from the line segment to the sphere’s center is $d=\left\|\vec{P_{1}O}+t\vec{P_{1}P_{2}}\right\|\leq r$, the path segment is deemed to collide with the obstacle. If collision is detected at any stage, add two control points at the path’s inflection point and refit the B-spline curve. Repeat this process until the entire path is collision-free.

To ensure trajectory feasibility, constraint verification must be performed after filtering kinematic parameters. The filtered joint velocities and accelerations are compared against the rated hardware limitations of the AUBO I5 robotic arm. Should any parameter exceed its bounds at any point in time, the acceleration in that region is reduced and refiltered. This iterative verification process continues until all kinematic parameters satisfy the hardware constraints.

#### 3.2.3. Kinematics parameter constraints and filtering.

Geometric smoothing only addresses the spatial form problem of the path. The movement of the robotic arm must also achieve motion stability based on kinematics through the control of speed and acceleration, that is, to avoid the inertial impact caused by sudden changes in the motion state, which leads to the liquid sloshing of the liquid in the volumetric flask. Therefore, it is necessary to establish the relationship between the parameters of the path’s parametric model and the speed, acceleration, and geometric parameters of the path. Through filtering and constraint strategies, the fluctuations in the movement can be eliminated.

Let the first-order derivative of the path parameter *u* (u∈[0,1]) with respect to time *t* be $\dot{u}=\frac{du}{dt}$ (the rate of parameter change), and *t*he second-order derivative be $\ddot{u}=\frac{d^{2}u}{dt^{2}}$ (the parameter acceleration). Then, the velocity (7) and acceleration (8) of the end effector can be derived from the geometric derivatives of the path:


$$v(t)=P^{\prime }(u)\cdot \dot{u}$$ (7)

$$a(t)=P^{\prime \prime }(u)\cdot {\dot{u}}^{2}+P^{\prime }(u)\cdot \ddot{u}$$ (8)

Here, $P^{\prime }(u)$ and $P^{\prime \prime }(u)$ represent the first derivative and the second derivative of a cubic B-spline curve, respectively. The speed is determined jointly by the geometric slope of the path ($P^{\prime }(u)$) and the rate of parameter change ($\dot{u}$). The acceleration consists of two parts: the centripetal acceleration caused by the path curvature ($P^{\prime \prime }(u)\cdot {\dot{u}}^{2}$) and the tangential acceleration caused by the change in the rate of parameter change ($P^{\prime }(u)\cdot \ddot{u}$).

To prevent liquid overflow, the peak velocity must be restricted while applying spline interpolation to the rate of change of the original parameters. This yields a continuous and differentiable $u^{\prime }(t)$; for example, during the transition of the path from a stationary to a moving phase, $u^{\prime }(t)$ gradually changes to a steady value following an S-shaped curve. This avoids abrupt step changes in velocity, ensuring that the maximum value of the first derivative of the velocity curve remains within permissible limits.

The sudden change in acceleration is the fundamental cause of the violent shaking of the liquid, and it must be filtered and smoothed. A third-order Butterworth low-pass filter is employed to filter the original acceleration curve, whose continuous-domain transfer function, as given by (9), is:


$$H(s)=\frac{1}{1+a_{1}s+a_{2}s^{2}+a_{3}s^{3}}$$ (9)

Here, *s* represents the Laplace operator, and the coefficients *a*_1_, *a*_2_, *a*_3_ are determined by the cutoff frequency *f*_*c*_ = 10Hz (to ensure the elimination of high-frequency vibration components). To adapt to the digital control system, it is transformed into the discrete domain through the bilinear transformation (10):


$$H(z)=\frac{b_{0}+b_{1}z^{-1}+b_{2}z^{-2}+b_{3}z^{-3}}{1+a_{1}z^{-1}+a_{2}z^{-2}+a_{3}z^{-3}}$$ (10)

Here, *z*^−1^ represents the unit delay operator, and the filtering coefficients *a*, *b* are calculated based on the sampling frequency *f*_*s*_ = 1000Hz.

The robotic arm achieves its final position through a series of joint movements from the base. Therefore, the velocity and acceleration of the end-effector must undergo motion decomposition, which converts them into corresponding joint motion parameters to avoid end-effector vibration induced by unreasonable velocity parameters at any single joint.

Based on an improved Denavit-Hartenberg (DH) method, a kinematic model is established where the joint angles $q=[q_{1},q_{2},\ldots ,q_{6}{]}^{T}$ and end-effector pose *P* satisfy the inverse kinematic relationship ***q*** = ikunc(*P*). By means of the Jacobian matrix ***J***(***q***), the end-effector velocity and acceleration can be converted into joint-space motion parameters, as expressed in (11) and (12).


$$\dot{q}=J^{-1}(q)\cdot v(t)$$ (11)

$$\ddot{q}=J^{-1}(q)\cdot \left(a(t)-\dot{J}(q,\dot{q})\cdot \dot{q}\right)$$ (12)

To ensure smooth coordination, the movement parameters of each joint need to be constrained. This prevents any single joint from experiencing excessive movement and excessive load, thereby ensuring the smoothness of the overall movement. The control is achieved through the above-mentioned speed and acceleration movement parameters, meeting the stability requirements of the volumetric flask for liquid handling.

## 4. Simulation experiments and results analysis

To verify the effectiveness of the VM-RRT* algorithm that integrates visual guidance sampling and multi-constraint optimization, this paper designs three sets of experiments for quantitative analysis: Firstly, a parameter sensitivity analysis is conducted, focusing on the noise coefficient as a key parameter, to explore its impact on the convergence efficiency and exploration ability of the algorithm, and to determine the optimal parameter configuration; Secondly, a comparison experiment of different path planning strategies is carried out, using indicators such as average planning time, path length, and sampling point number to compare the efficiency and path quality of VM-RRT* with other commonly used algorithms; Thirdly, a simulation comparison experiment of mechanical arm smoothing strategies is conducted to observe the influence of different smoothing methods on the stability and trajectory continuity of the mechanical arm movement after VM-RRT* generates the basic path, ensuring a safe and smooth grasping process.

### 4.1. Experimental environment, detection model and parameter configuration

The experiment was conducted under the Windows 11 operating system, using an AMD Ryzen 7 6800H processor and 16 GB of RAM. The algorithm simulation was completed using the MATLAB R2022b software, and the mechanical arm model selected was the 6-degree-of-freedom AUBO I5.

The experimental scenario of this study corresponds to the actual laboratory operation area: with a multi-layer metal experimental bench as the core carrier, experimental equipment such as beakers and droppers were randomly placed on the bench as interference objects. Most samples were in the situation where the target container was obscured by the bench or the equipment. The scene included complex factors such as light changes, background interference and occlusion.

Visual detection used the improved YOLOv11 algorithm (PC-YOLO), and the model training dataset contained 1247 real-scene images. Before training, data augmentation was completed through strategies such as brightness adjustment and angle rotation. PC-YOLO relied on the C3K2_PPA module to improve the detection effect of small targets, and the CMUNeXt Block module to enhance the ability to extract global information. Combined with the MPDIoU loss function to optimize the accuracy and training convergence speed of bounding box regression, it can well adapt to the complex detection environment of the laboratory. Table 1 compares the performance of different target detection algorithms. Compared with other models, PC-YOLO shows higher performance in terms of precision, recall rate, and mAP.

**Table 1:** Performance comparison table of different object detection algorithms.

Model Name	Precision/%	Recall/%	mAP50/%	mAP50-95/%
YOLOv8n	86.7	94.5	91.4	84.5
YOLOv10n	87.3	95.5	91.7	80.3
YOLOv11n	86.9	95.5	92.2	81.2
PC-YOLO	88.8	95.7	94.2	85.0

**Fig 8:**
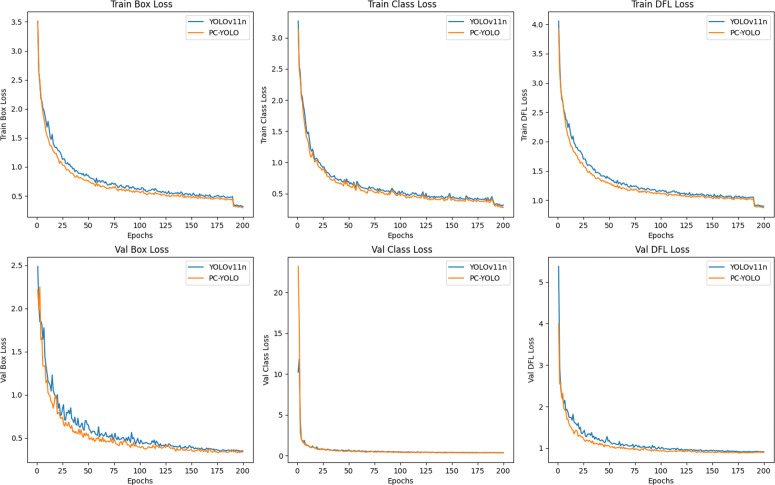
Comparison of evaluation metrics between YOLOv11n and PC-YOLO.

Fig 8 illustrates the evolution of four metrics—precision, recall, mAP50, and mAP50-95—during the training process of PC-YOLO and YOLOv11n. The horizontal axis represents the number of training epochs, while the vertical axis denotes the metric values. Experimental results demonstrate that PC-YOLO outperforms YOLOv11n across all metrics. Specifically, mAP50 improves from 92.2% to 94.2%, while mAP50-95 increases from 81.2% to 85%. This represents a significant enhancement in detection performance under high IoU standards, particularly within complex environments.

**Fig 9:**
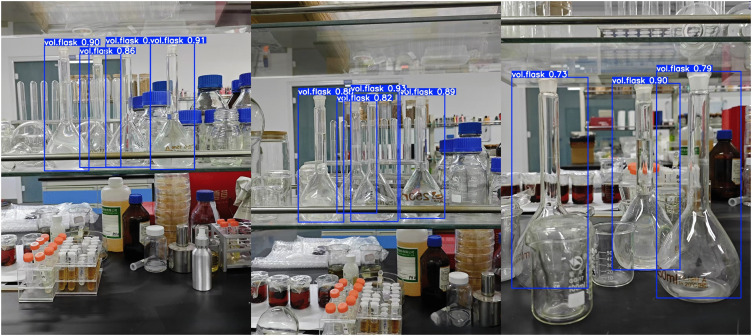
YOLOv11n detection result image.

**Fig 10:**
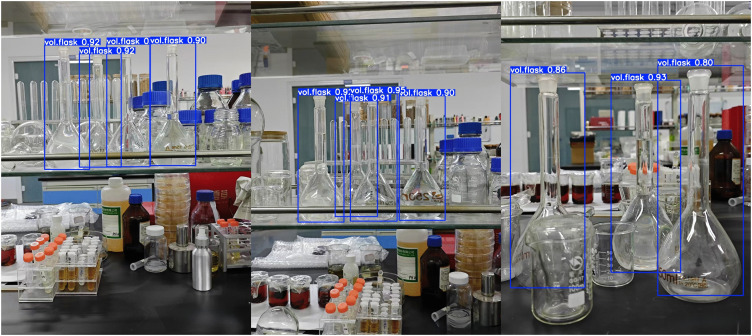
PC-YOLO detection result image.

Figs 9 and 10 respectively display the detection results of YOLOv11n and PC-YOLO. YOLOv11n exhibits defects in bounding box offset and overlap, while PC-YOLO demonstrates more precise bounding box positioning that closely aligns with the target, along with higher overall confidence scores, resulting in greater reliability in target recognition.

In the kinematic modeling of the robotic arm, the standard DH parameters, which include the joint angle $\theta_{i}$, the link offset *d*_*i*_, the twist angle $\alpha_{i}$, and the link length *a*_*i*_, are the core parameters that describe the spatial geometric relationship between each joint and the link. The standard DH parameter table of the AUBO I5, as a 6-degree-of-freedom rotational joint collaborative robot, is shown in Table 2.

**Table 2:** AUBO I5 robotic arm standard DH parameter table.

Joint *i*	$\theta_{i}/({\;}^{\circ })$	$d_{i}/\text{mm}$	$\alpha_{i}/({\;}^{\circ })$	$a_{i}/\text{mm}$
1	*θ*_*1*_	121.8	0	0
2	*θ*_*2*_	0	−90	408.0
3	*θ_3_*	0	0	376.0
4	*θ_4_*	121.3	−90	0
5	*θ_5_*	0	90	0
6	*θ_6_*	93.8	−90	0

**Fig 11:**
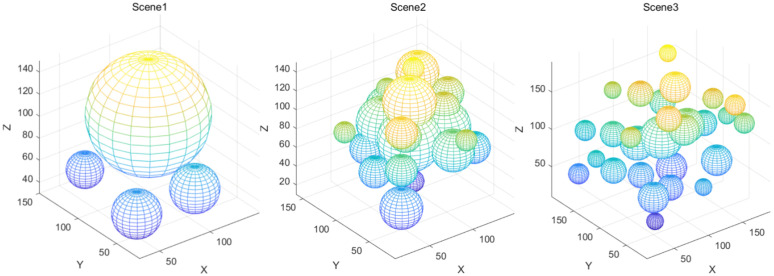
Schematic diagram of obstacle distribution in three simulation scenarios.

Fig 11 depicts three distinct obstacle simulation scenarios designed in this study to validate the performance of path planning algorithms, each testing different dimensions of algorithmic behaviour. The left image depicts a low-density, unobstructed core path scenario, designated as Scene1. Only a few obstacles are placed at the periphery of the operational space to test the algorithm’s fundamental planning efficiency under low-interference conditions. The middle image shows a high-density core path with full obstruction scenario, designated as Scene2. Obstacles are concentrated along the core route between the target container and the starting point, specifically designed to validate the algorithm’s obstacle avoidance and detour capabilities when the critical path is completely blocked. The right diagram depicts a scattered and disordered obstacle scenario, designated as Scene 3. Obstacles are randomly dispersed throughout the entire operational space, with both dimensions and positions distributed haphazardly. This tests the algorithm’s path planning stability amidst chaotic obstacle distribution and complex spatial interference. These three scenarios encompass typical obstacle challenge types encountered in path planning tasks, validating the algorithm’s performance under varying obstacle characteristics.

The maximum sampling times for all different algorithms are set to 10000 times, with a sampling step size of 50 mm, a neighborhood search radius of 550 mm, a target detection confidence level of 0.8, and the number of control points in cubic B-spline fitting is 1.5 times that of path points; in the speed and acceleration optimization steps, the cutoff frequency of the 3rd-order Butterworth low-pass filter is 10 Hz, and the sampling frequency is 1000 Hz.

In this study, the neighborhood search radius for the rerouting phase is fixed at 550 mm. This value is tailored to the confined spatial scale of the volumetric flask retrieval scenario and is designed to align with the 50 mm sampling step size. This approach ensures that there are sufficient nodes within the neighborhood to support rerouting optimization while avoiding the inclusion of invalid nodes and increased computational load that would result from an excessively large radius, thereby achieving a balance between neighborhood search efficiency and path optimization performance. This parameter design represents a reasonable compromise between the asymptotic optimality theory of the RRT* algorithm and the real-time requirements of path planning. The strict asymptotic optimality of the classical RRT* algorithm requires that the rerouting radius adaptively decrease with the number of nodes in the path tree. Although the fixed-radius design adopted in this study sacrifices strict asymptotic optimality at the theoretical level, through the coordinated optimization of vision-guided hybrid sampling and multi-constraint path smoothing, it enables the planned path to achieve quasi-optimal performance in the bottle-picking scenario, while simultaneously reducing computational complexity and enhancing the real-time performance and convergence stability of the algorithm.

### 4.2. Hyperparameter sensitivity analysis

In the research of path planning and sampling strategies, the noise coefficient is a key parameter that affects the convergence efficiency and exploration ability of the algorithm. This section takes Scenario One as the experimental environment and designs a multi-confidence cross-validation experiment, combining the obtained experimental data to explore the specific impact of the noise coefficient on the algorithm performance.

This path planning hyperparameter sensitivity experiment is built on the MATLAB platform, setting each group of parameters to be repeated 50 times for experimental results to ensure reliability. The experiment selects six noise coefficients of 0.05, 0.1, 0.15, 0.2, 0.25, and 0.3 and four confidence levels of 0.6, 0.7, 0.8, and 0.9 for cross-validation.

From Tables 3, 4, 5, and 6, it can be concluded that the comprehensive performance of the noise coefficient of 0.2 is the most prominent. In terms of planning time, its average planning time across most confidence level scenarios is concentrated in the range of 3.2 to 3.7 seconds; for example, at a confidence level of 0.8, it is only 3.22 seconds, which is relatively short among all noise coefficients. The path length always remains stable within the reasonable range of 295–298, with no significant fluctuations, while the number of sampling points mostly stays between 286 and 332, balancing exploration comprehensiveness and sampling efficiency. Compared with other noise coefficients, 0.05 and 0.1 exhibit longer planning times under certain confidence levels, whereas 0.25 and 0.30 show significantly higher planning times and sampling point counts in some confidence scenarios.

**Table 3:** Effect of noise coefficient on path planning indicators at confidence 0.6.

Noise Coefficient	Time/s	Path Length/mm	Sampling Points	Success Rate/%
0.05	3.48	296.92	311.50	100
0.1	3.19	296.66	287.98	100
0.15	3.32	295.66	294.80	100
0.2	3.69	297.68	332.38	100
0.25	3.28	299.90	294.26	100
0.3	3.44	296.83	311.80	100

**Table 4:** Effect of noise coefficient on path planning indicators at confidence 0.7.

Noise Coefficient	Time/s	Path Length/mm	Sampling Points	Success Rate/%
0.05	3.75	293.88	340.48	100
0.1	3.23	295.61	290.14	100
0.15	3.23	295.52	294.44	100
0.2	3.52	298.32	316.02	100
0.25	3.42	297.37	313.02	100
0.3	3.72	296.65	332.62	100

**Table 5:** Effect of noise coefficient on path planning indicators at confidence 0.8.

Noise Coefficient	Time/s	Path Length/mm	Sampling Points	Success Rate/%
0.05	3.26	296.83	288.40	100
0.1	3.18	297.30	285.32	100
0.15	3.60	295.58	322.22	100
0.2	3.22	294.98	286.64	100
0.25	3.29	296.99	298.88	100
0.3	3.56	294.78	319.42	100

**Table 6:** Effect of noise coefficient on path planning indicators at confidence 0.9.

Noise Coefficient	Time/s	Path Length/mm	Sampling Points	Success Rate/%
0.05	3.29	296.57	289.92	100
0.1	3.18	298.85	281.06	100
0.15	3.67	294.07	321.02	100
0.2	3.67	296.87	321.98	100
0.25	3.95	297.37	349.18	100
0.3	3.98	294.59	344.82	100

Therefore, in this paper, a noise coefficient of 0.2 is chosen as the parameter for the hybrid sampling strategy. This choice not only meets the stable convergence requirements under low confidence levels, but also effectively balances exploration capability under high confidence environments.

### 4.3. Path planning algorithm comparison experiments and results analysis

To validate the performance differences of various path planning algorithms in complex environments, four algorithms were selected: RRT*, Bi-RRT, Artificial Potential Field-RRT* (APF-RRT*), and the proposed VM-RRT*. Under uniform experimental conditions that include three types of differentiated obstacle simulation scenarios (Scene 1, Scene 2, and Scene 3) and a target detection confidence level of 0.8, 50 simulation trials were conducted for each algorithm. All algorithms achieved a 100% planning success rate throughout the experiments. Comparisons were made based on three metrics: average planning time, average path length, and average number of sampling points. The results are presented in Table 7.

**Table 7:** Comparison of different path planning algorithms.

Algorithm	Index	Scene1	Scene2	Scene3
RRT*	Time / s	4.0	4.3	3.9
	Length / mm	295.4	287.2	278.4
	Number	357.7	425.0	326.2
**Bi-RRT**	Time / s	1.3	1.4	1.2
	Length / mm	387.0	381.2	367.0
	Number	287.4	162.7	127.4
APF-RRT*	Time / s	2.7	8.4	2.5
	Length / mm	288.1	283.0	274.9
	Number	379.0	417.0	321.0
VM-RRT*	Time / s	3.3	3.3	3.2
	Length / mm	297.2	293.6	278.0
	Number	298.4	337.3	257.7

**Fig 12:**
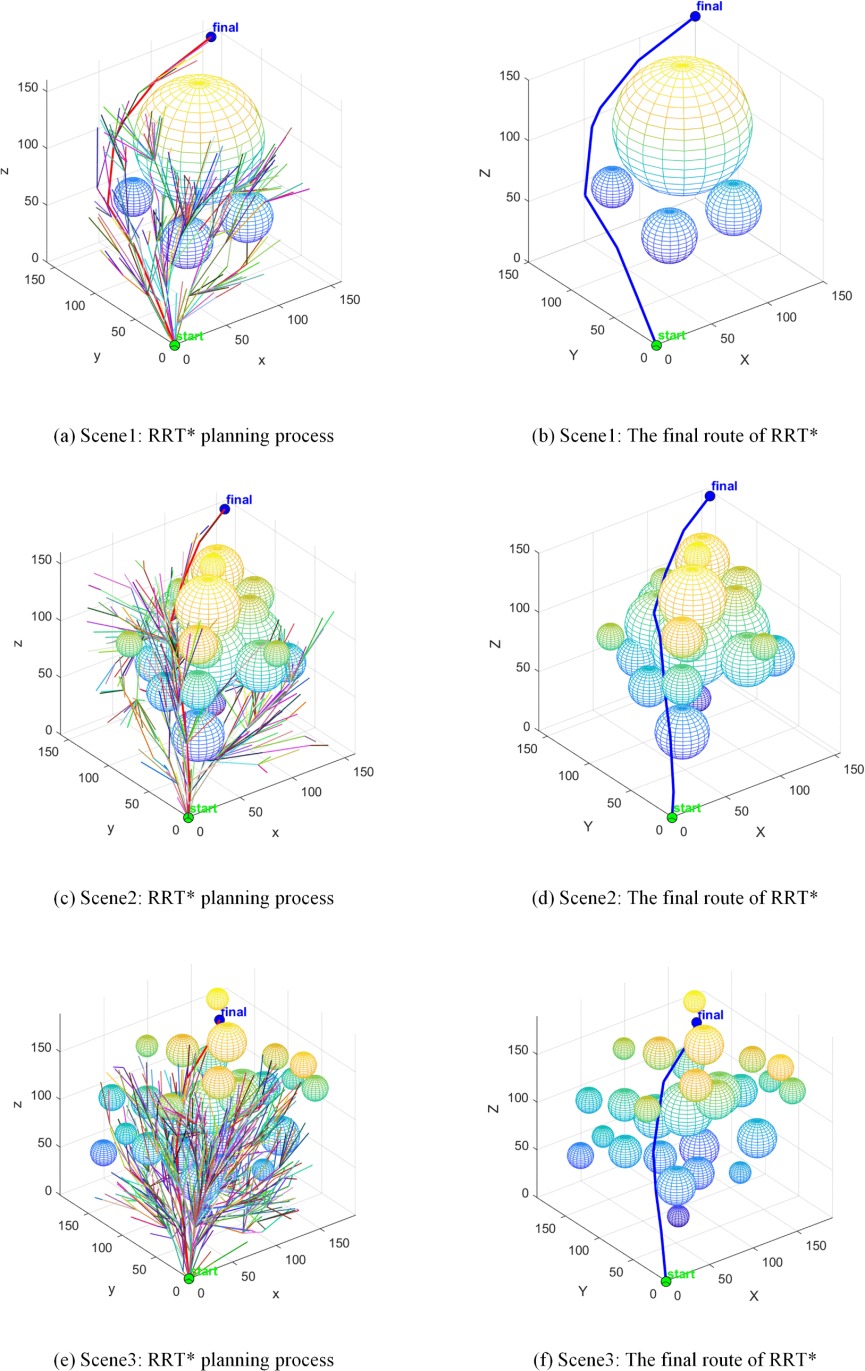
RRT* path planning.

The RRT* algorithm employs a global random sampling strategy, resulting in a search tree characterised by broad and sparse branching, as illustrated in Fig 12. Whilst this sampling approach ensures the path’s progressive optimality with no significant path length redundancy, its inherent randomness substantially elevates planning computational costs. In the sparsely obstructed Scene1 alone, the algorithm consumed 4.0 seconds. In the more densely obstructed Scene2, the number of sampling points reached 425, rendering it inefficient and impractical for real-world applications.

**Fig 13:**
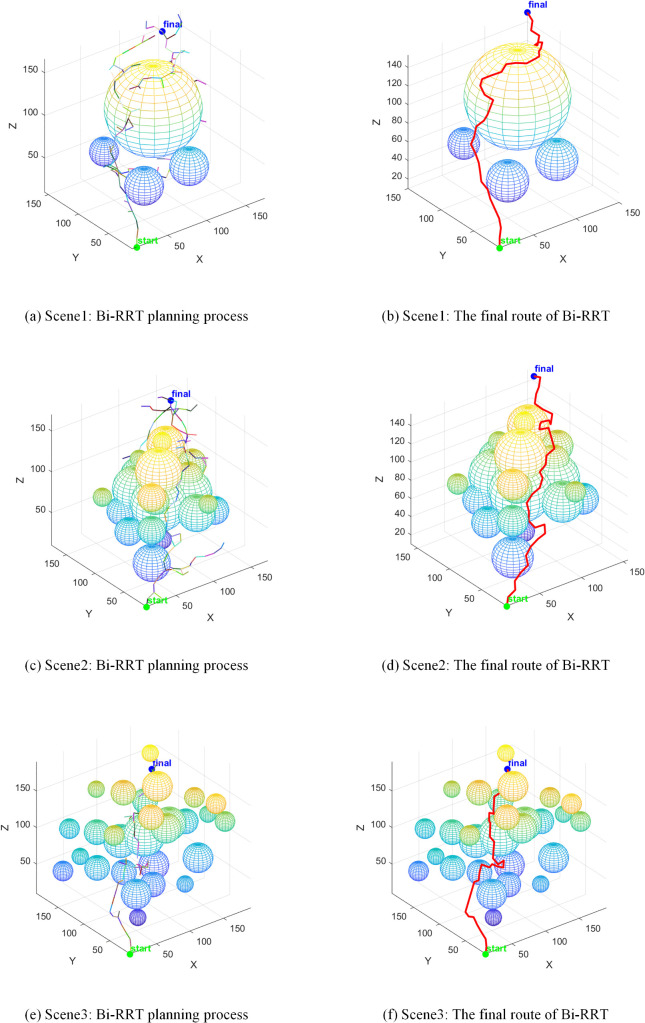
Bi-RRT path planning.

The Bi-RRT algorithm employs a bidirectional expansion strategy to reduce planning time for Scene1 to 1.3 seconds. However, this characteristic of rapid convergence from both start and end points sacrifices the algorithm’s global path optimisation capability, as illustrated in Fig 13. The path length generated by this algorithm in Scene1 reached 387.0 mm, with over 30% comprising ineffective paths. This fundamentally represents a design flaw where the algorithm trades path quality for planning speed, significantly increasing collision risks during robotic arm operations and failing to meet safety requirements for precision tasks.

**Fig 14:**
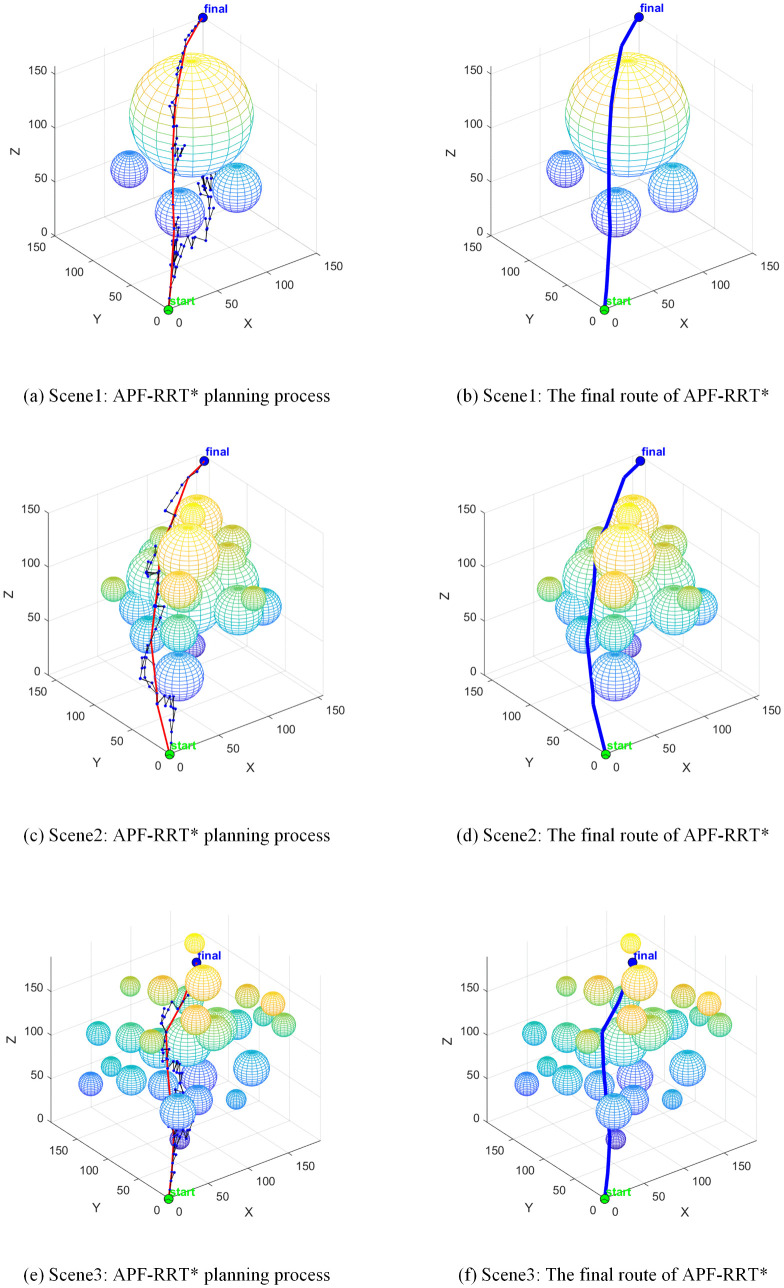
APF-RRT* path planning.

The APF-RRT* algorithm integrates the attraction-repulsion guidance mechanism of the artificial potential field method. In simple scenes such as Scene1 and Scene3, planning times were controlled at 2.7 seconds and 2.5 seconds respectively, demonstrating a marked improvement in sampling efficiency. However, in the densely obstructed Scene 2, mutual interference in repulsive directions induces local path oscillations, as evidenced by the meandering segments in Fig 14. Consequently, planning time escalated to 8.4 seconds with 417 sampling points, revealing the algorithm’s limitations in adapting to complex operational conditions.

**Fig 15:**
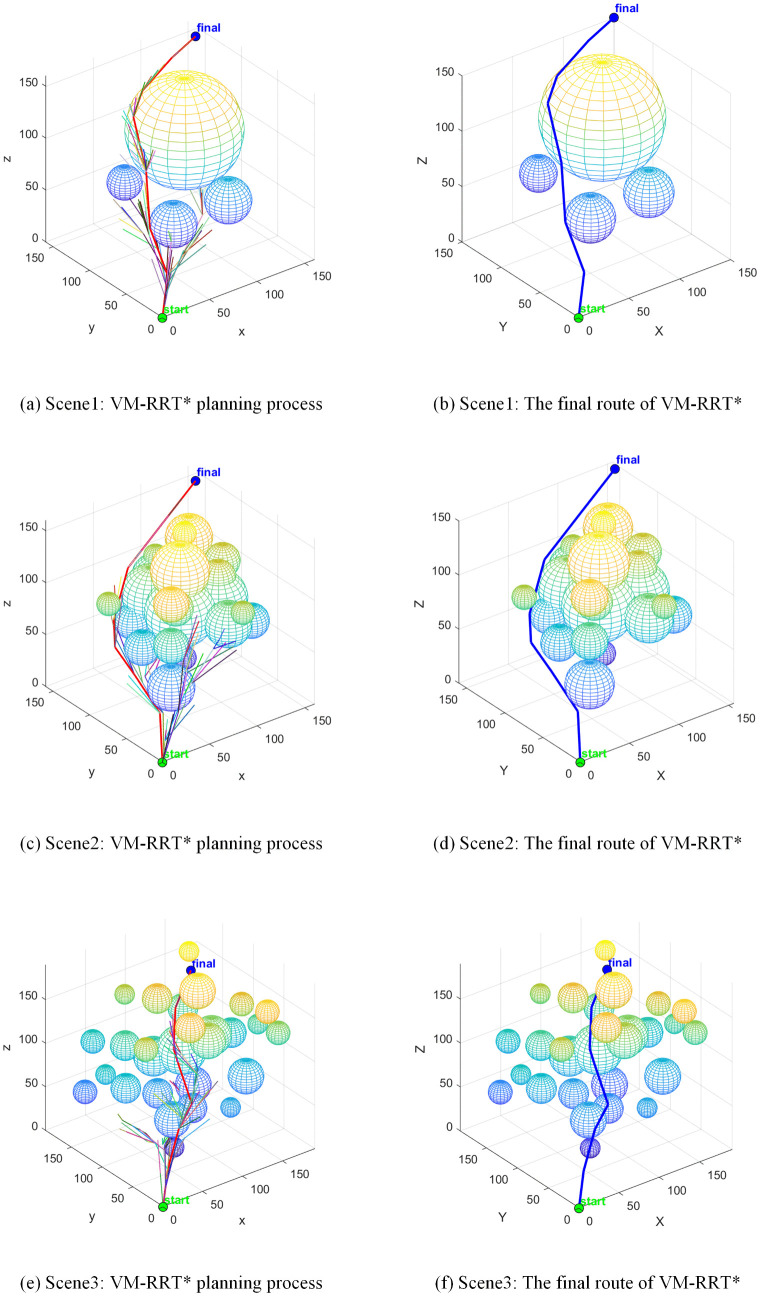
VM-RRT* path planning.

The VM-RRT* algorithm proposed in this paper is shown in Fig 15. Its search tree branches are more concise and it avoids the path defects of Bi-RRT. In Scene1, this algorithm takes 3.3 seconds, which is 17.5% shorter than RRT*. The number of sampling points is 298.4, which is 16.6% less than RRT*. At the same time, this algorithm can approach the shortest path length and effectively avoids the scene adaptability and safety defects of similar algorithms.

Combining box plot data, we analyse the stability and standard deviation performance of RRT* and VM-RRT*. The blue box in the box plot reflects data fluctuation amplitude and algorithm stability, the red median line corresponds to the median performance level, the black line represents the normal fluctuation boundary of performance, and the red + sign indicates performance outliers.

**Fig 16:**
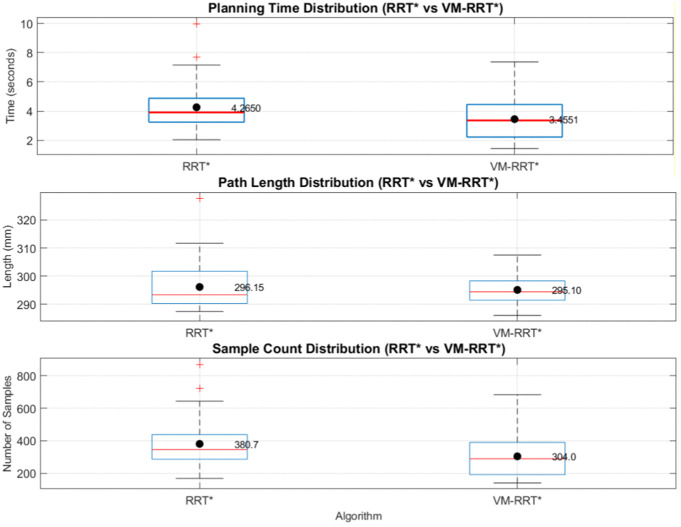
Comparison of metric distributions between RRT* and VM-RRT* (Scene 1).

In Scene 1, as illustrated in Fig 16, VM-RRT* reduces planning time by approximately 22.6% and the number of sampling points by approximately 20.2% compared to RRT*, while maintaining comparable path length. The tighter box plot and error line visually indicate lower data dispersion.

**Fig 17:**
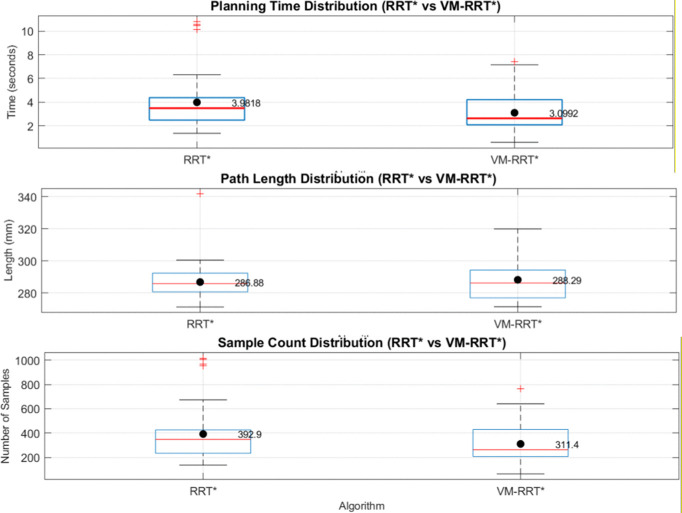
Comparison of metric distributions between RRT* and VM-RRT* (Scene 2).

In Scene 2, as depicted in Fig 17, planning time and sampling points were reduced by approximately 22.2% and 20.7% respectively compared to RRT*. Path length was slightly shorter than that of RRT*, with no significant outliers observed in the core metrics of planning time and sampling points. The contraction trend in the box plot and error bars indicates a smaller standard deviation than RRT*, reflecting narrower data fluctuation ranges.

**Fig 18:**
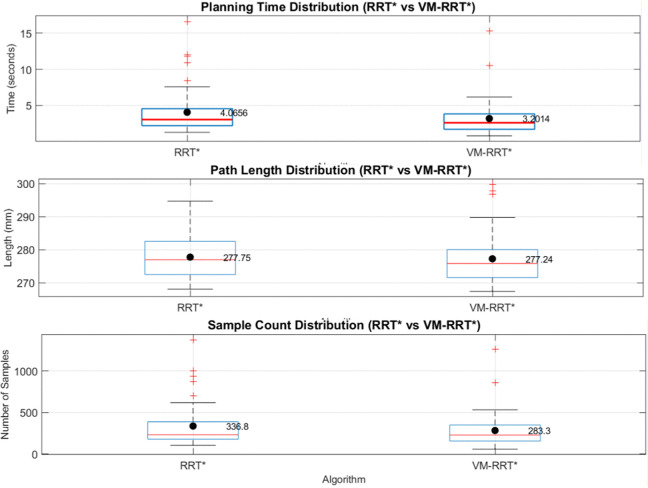
Comparison of metric distributions between RRT* and VM-RRT* (Scene 3).

In Scene 3, as illustrated in Fig 18, planning time and the number of sampling points decreased by approximately 21.3% and 15.9% respectively compared to RRT*. Path length remained virtually identical to RRT*, while the compactness of its box plot and error bars far exceeded that of RRT*, indicating a significantly smaller standard deviation in performance metrics and substantially reduced data fluctuation.

Across diverse testing scenarios, VM-RRT* consistently achieves a marked improvement in planning efficiency. Concurrently, its more compact enclosure and narrower error margin continually demonstrate reduced standard deviation and lower data volatility.

### 4.4. Simulation comparison and validation of trajectory smoothing Strategies

**Fig 19:**
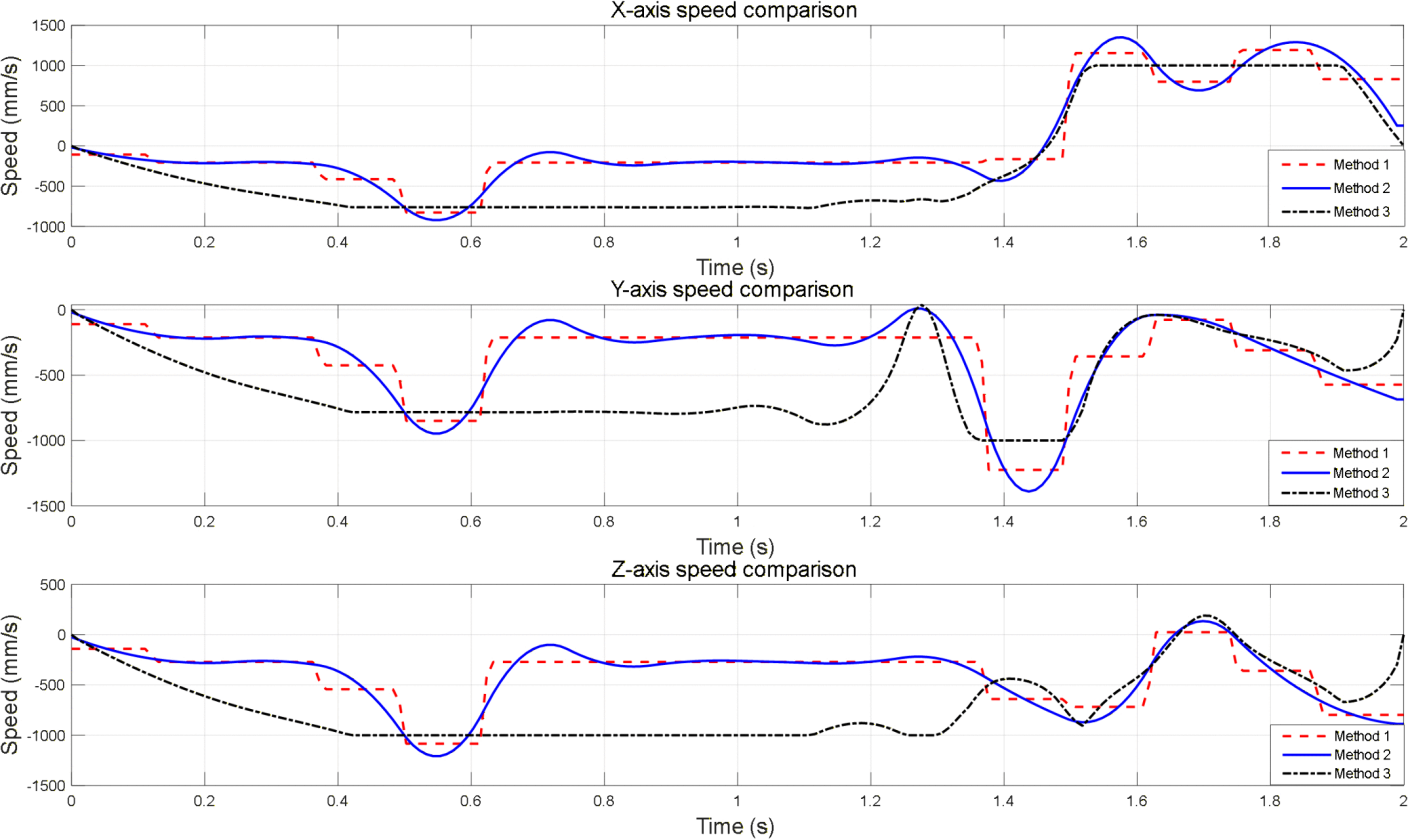
Comparison of three-Axis velocity curves for robotic arms under different smoothing strategies.

**Fig 20:**
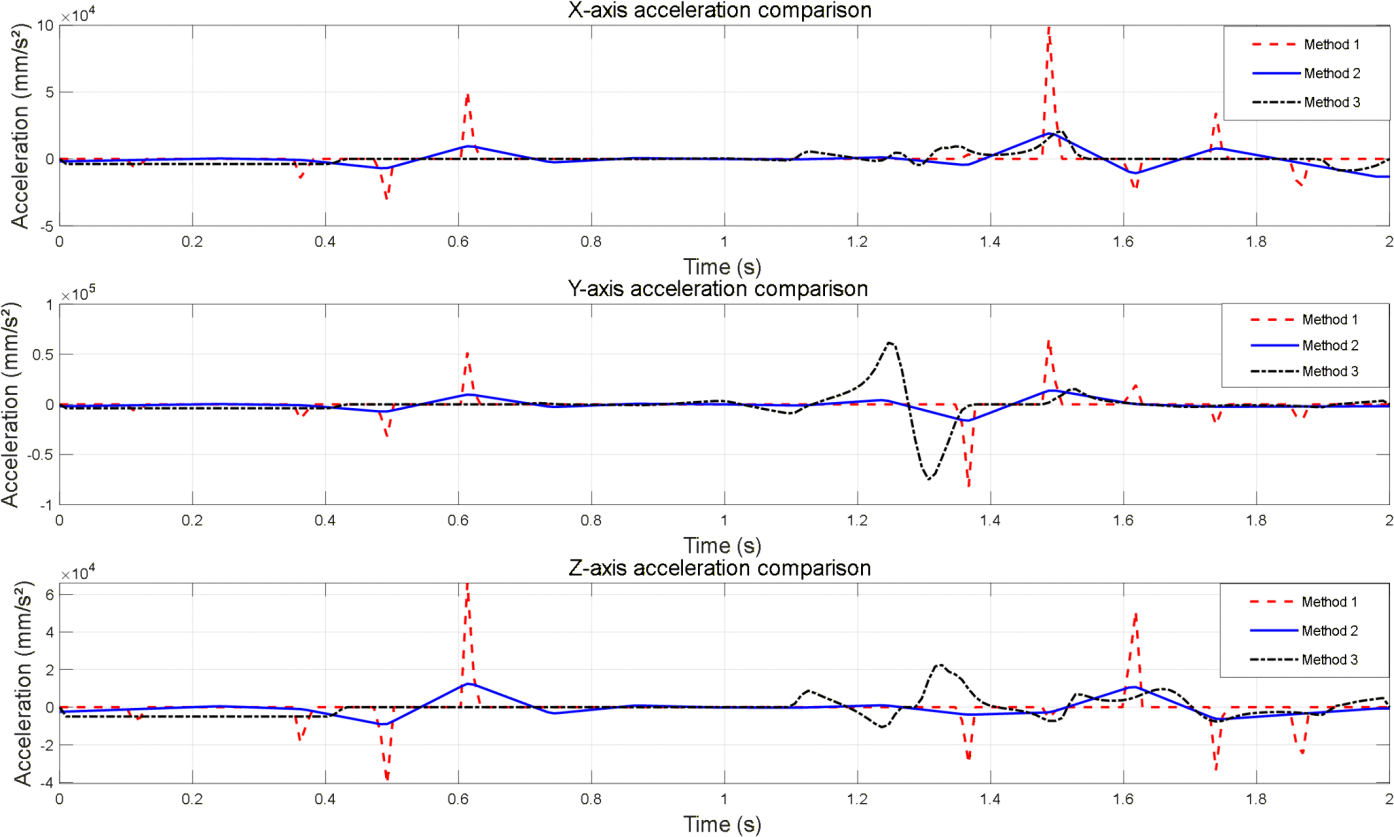
Comparison of three-Axis acceleration curves for robotic arms under different smoothing strategies.

To validate the impact of different smoothing strategies on robotic arm motion performance, a baseline trajectory was generated using the VM-RRT^*^ vision-guided sampling target orientation mechanism within Scenario 1. Three experimental methods were established, differing solely in their post-processing stages, with results analysed concurrently. The experiment utilised the MATLAB Robotics Toolbox to construct an AUBO I5 robotic arm model. Trajectory continuity and operational smoothness were compared from a kinematic curve perspective. Comparisons of velocity and acceleration across the three axes for each method are presented in Figs 19 and 20.

Method One performs only basic processing on discrete path points, employing coordinate concatenation and linear transitions combined with Savitzky-Golay filtering for preliminary smoothing. Velocity and acceleration are calculated via first- and second-order differentiation without incorporating trajectory optimisation or joint constraints. Its velocity curve exhibits step-like jumps, while the acceleration curve displays high-frequency sawtooth fluctuations, failing to meet the smoothness requirements for precision operations.

Method Two employs cubic B-spline fitting to optimise path curvature while imposing maximum joint velocity constraints to trim out-of-bounds values. Combining spline interpolation with Butterworth low-pass filtering for velocity and acceleration processing yields smooth, arc-shaped motion parameter curves. All parameters remain within hardware-supported limits, balancing smoothness and safety to meet precision operation requirements.

Method Three employs the Time-Optimal Path Rate-Constrained Parametrisation (TOPP-RA) algorithm as a baseline for global optimisation of the fundamental path. Whilst achieving trajectory optimisation, this approach suffers from locally abrupt changes in velocity and acceleration curves due to stringent hardware constraints, resulting in poorer smoothness and practical applicability compared to Method Two.

**Fig 21:**
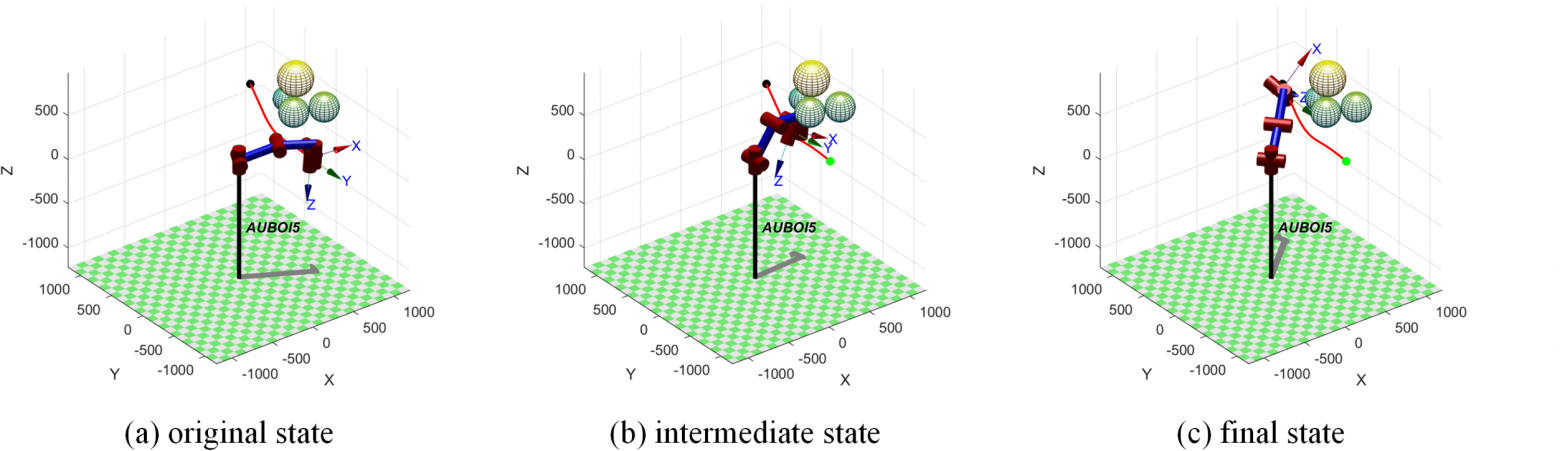
Simulation diagram of the movement process of the robotic arm.

The simulation of the robotic arm’s movement process is shown in Fig 21. From the initial posture to the intermediate obstacle avoidance state, the end effector moves around the obstacle in a continuous curve.

## Conclusion

This paper addresses the path planning problem for robotic arms manipulating volumetric flasks in chemical laboratories by proposing the VM–RRT^*^ planning algorithm. This approach resolves the shortcomings of the RRT^*^ algorithm, namely its slow convergence speed and poor path smoothness. The algorithm acquires the volumetric flask’s spatial coordinates through target detection and employs dynamic bias sampling to avoid sampling invalid regions, thereby accelerating convergence within the target area. Experimental results demonstrate that VM-RRT^*^ achieves an average planning time of 3.27 seconds, representing a reduction of approximately 20% compared to RRT^*^’s 4.07 seconds, thereby effectively enhancing operational efficiency. Concurrently, the planned path undergoes triple B-spline fitting alongside velocity, acceleration, and kinematic filtering. This achieves dual path smoothing at both geometric and kinematic levels, preventing abrupt changes in end-effector velocity and acceleration. Consequently, the liquid remains stable during flask handling, enhancing operational safety. Limitations of this algorithm include: when obstacles densely surround the target, dynamic offset sampling may cause excessive concentration of samples in the target area. This can lead to sampling stuttering near obstacles, reducing planning efficiency. Future research will explore real-time path adjustment strategies near obstacles to accommodate complex laboratory operational requirements.

## Supporting information

S1 File Data and code used in this article. This file contains MATLAB-related code and experimental data to reproduce the results presented in the manuscript.
(ZIP)
