# Supplementary material for: Path planning for volumetric flask grasping based on visual guidance and multi-constraint optimization
Source: PLoS One. 2026 Apr 20;21(4):e0347043. doi: 10.1371/journal.pone.0347043 (PMC13095110; doi:10.1371/journal.pone.0347043)
Supplement: S1 File — This file contains MATLAB-related code and experimental data to reproduce the results presented in the manuscript. (ZIP) [file pone.0347043.s001.zip › 支持信息/apfrrtstar.pdf]

```
function [path, T, sampleCount] = apfrrtstar(start_point, goal_point, obstacles, fig_handle)
% APF-RRT* 3D 路径规划核心函数
% 输入：
%     start_point/goal_point - 起点/终点 [x,y,z]
%     obstacles - 障碍物列表
%     fig_handle - 过程图的 figure 句柄（用于绘制过程节点）
% 输出：
%     path - 最终路径矩阵（n×3）
%     T - RRT 树结构
%     sampleCount - 总采样点数

% 初始化参数
max_iter = 10000;              % 最大迭代次数
step_size = 10;                % 扩展步长
goal_tolerance = 10;           % 目标容忍度 8
radius = 50;                   % 重连半径 20
sampleCount = 0;               % 采样数初始化
path = [];                     % 路径初始化

% APF 参数
alpha = 1.2;   % 目标吸引力权重（增大，优先向目标走）
beta   = 0.3;   % 随机点权重（减小）
k_rep = 80;     % 斥力系数（增大，避障更明显）
rep_range = 50;% 斥力生效范围

% 初始化 RRT 树
T = struct('node', start_point, 'parent', -1);
x_max = 200; y_max = 200; z_max = 200;

% 主循环
for iter = 1:max_iter
    % 1. 生成随机采样点
    if rand < 0.2   % 20%概率采样目标点，加速收敛
        rand_point = goal_point;
    else
        rand_point = rand(1,3) * 200;   % 场景内随机采样
    end
    sampleCount = sampleCount + 1;   % 累加采样数

    % 2. 找到离随机点最近的节点
    min_dist = inf;
    nearest_idx = 1;
    for i = 1:length(T)
        dist = norm(T(i).node - rand_point);
```

```
        if dist < min_dist
            min_dist = dist;
            nearest_idx = i;
        end
    end
    nearest_node = T(nearest_idx).node;

    % 3. 计算 APF 合力方向
    % 吸引力（目标点+随机点）
    vec_goal = goal_point - nearest_node;
    if norm(vec_goal) < 1e-6
        att_goal = [0,0,0];
    else
        att_goal = alpha * (vec_goal / norm(vec_goal));
    end

    vec_rand = rand_point - nearest_node;
    if norm(vec_rand) < 1e-6
        att_rand = [0,0,0];
    else
        att_rand = beta * (vec_rand / norm(vec_rand));
    end

    % 斥力（障碍物）
    rep_force = [0,0,0];
    for i = 1:length(obstacles)
        obs = obstacles{i};
        obs_center = obs(1:3);
        r = obs(4);
        dist_obs = norm(nearest_node - obs_center);
        effective_dist = dist_obs - r;

        if (effective_dist > 0) && (effective_dist < rep_range)
            direction = (nearest_node - obs_center) / dist_obs;
            mag = k_rep * (1/effective_dist - 1/rep_range) / (effective_dist^2);
            rep_force = rep_force + mag * direction;
        end
    end

    % 总合力
    total_force = att_goal + att_rand + rep_force;
    if norm(total_force) < 1e-6    % 合力为 0 时随机方向
        total_force = randn(1,3);
    end
```

```
    direction = total_force / norm(total_force);

    % 4. 生成新节点
    new_node = nearest_node + direction * step_size;
    % 边界检查（限制在场景内）
    new_node(new_node < 0) = 0;
    new_node(new_node > 200) = 200;

    % 5. 碰撞检测（无碰撞才扩展）
    if ~checkPath3(nearest_node, new_node, obstacles)
        % 添加新节点到树
        T(end+1) = struct('node', new_node, 'parent', nearest_idx);

        % 6. 绘制过程节点（蓝色小点）和分支（黑色细线）
        figure(fig_handle); hold on;
        plot3(new_node(1), new_node(2), new_node(3), 'bo', 'MarkerSize', 2, 'MarkerFaceColor', 'b');
        parent_node = T(nearest_idx).node;
        line([parent_node(1), new_node(1)], [parent_node(2), new_node(2)], [parent_node(3), new_node(3)], 'Color', 'k', 'LineWidth', 0.5);

        % 7. RRT* 重连优化
        for i = 1:length(T)-1
            if i ~= nearest_idx && norm(T(i).node - new_node) < radius
                if ~checkPath3(T(i).node, new_node, obstacles)
                    % 计算新路径成本
                    cost_old = path_cost(T, length(T));
                    cost_new = path_cost(T, i) + norm(T(i).node - new_node);
                    if cost_new < cost_old
                        T(end).parent = i;   % 更新父节点
                    end
                end
            end
        end

        % 8. 检查是否到达目标
        if norm(new_node - goal_point) < goal_tolerance
            % 回溯路径
            path = extract_path(T, length(T));
            path = [path; goal_point];   % 添加终点
            break;  % 找到路径，终止迭代
        end
    end
end
```

```
% 容错：迭代结束未找到路径
if isempty(path) && iter >= max_iter
    disp('APF-RRT*：未到达目标点，建议调整障碍物或参数');
end
end

%% ========================== 子 函 数 1 ： 路 径 成 本 计 算 ==========================
function cost = path_cost(T, idx)
cost = 0;
current = idx;
while T(current).parent ~= -1
    parent = T(current).parent;
    cost = cost + norm(T(current).node - T(parent).node);
    current = parent;
end
end

%% ========================== 子 函 数 2 ： 提 取 路 径 ==========================
function path = extract_path(T, idx)
path = [];
current = idx;
while current ~= -1
    path = [T(current).node; path];
    current = T(current).parent;
end
end

%% ========================== 子 函 数 3 ： 3D 线 段 - 球 体 碰 撞 检 测 ==========================
function collision = checkPath3(p1, p2, obstacles)
collision = false;
p1 = p1(:).'; p2 = p2(:).';
d = p2 - p1;

for i = 1:length(obstacles)
    obs = obstacles{i};
    if length(obs) == 4
        center = obs(1:3); r = obs(4);
        f = p1 - center;
        a = dot(d, d);
        b = 2 * dot(f, d);
```

```
            c = dot(f, f) - r^2;
            discriminant = b^2 - 4*a*c;

            if discriminant >= 0
                sqrtD = sqrt(discriminant);
                t1 = (-b - sqrtD) / (2*a);
                t2 = (-b + sqrtD) / (2*a);
                % 线段与球体相交（t 在[0,1]范围内）
                if (t1 >= 0 && t1 <= 1) || (t2 >= 0 && t2 <= 1)
                    collision = true;
                    return;
                end
            end
        end
    end
end
```
