# Supplementary material for: Path planning for volumetric flask grasping based on visual guidance and multi-constraint optimization
Source: PLoS One. 2026 Apr 20;21(4):e0347043. doi: 10.1371/journal.pone.0347043 (PMC13095110; doi:10.1371/journal.pone.0347043)
Supplement: S1 File — This file contains MATLAB-related code and experimental data to reproduce the results presented in the manuscript. (ZIP) [file pone.0347043.s001.zip › 支持信息/bi_RRTmain.pdf]

```
clc;
clear;
close all;
warning off;
totalTime = 0;
pathLength = 0;
sampleCount = 0;

try
    [totalTime, pathLength, sampleCount] = runSingleBiRRT();
    fprintf('单次实验成功 | 时间: %.4fs | 路径长度: %.2f | 采样点数: %d\n', ...
        totalTime, pathLength, sampleCount);
catch ME
    fprintf('单次实验失败 | 原因: %s\n', ME.message);
end

%% 显示统计结果
disp('=== 双向 RRT 路径规划结果 ===');
fprintf('规划时间 = %.4f 秒 \n', totalTime);
fprintf('路径长度 = %.2f \n', pathLength);
fprintf('采样点数 = %d \n', sampleCount);
disp('========================');

%% 定义单次路径规划函数
function [totalTime, pathLength, sampleCount] = runSingleBiRRT()
    %% 绘制障碍物(以球为例，方便计算)
    circleCenter = [100,100,100;50,50,50;100,40,60;60,130,50];
    r = [50;20;20;15];    % 半径
% circleCenter = [
%        80,    80,    80;      % 球体 1：中心区域大球
%        80,    80,    120;     % 球体 2：中心区域上方球
%        80,    120,   80;      % 球体 3：中心区域右侧球
%        120,   80,    80;      % 球体 4：中心区域前方球
%        40,    40,    40;      % 球体 5：左侧群大球
%        40,    40,    80;      % 球体 6：左侧群中间球（Z 方向）
%        40,    40,    120;     % 球体 7：左侧群上方球（Z 方向）
%        40,    80,    60;      % 球体 8：左侧群右侧球（Y 方向）
%        120,   120,   120;     % 球体 9：右侧群大球
%        120,   120,   80;      % 球体 10：右侧群中间球（Z 方向）
%        120,   120,   40;      % 球体 11：右侧群下方球（Z 方向）
%        120,   80,    100;     % 球体 12：右侧群左侧球（Y 方向）
%        60,    120,   60;      % 球体 13：上方群左侧球
%        100,   140,   80;      % 球体 14：上方群大球（Y 方向突出）
%        140,   100,   100;     % 球体 15：上方群右侧球
```

```
%         60,     60,     100;     %  球体 16:  下方群左侧球
%         100,   40,     80;       %  球体 17:  下方群大球（Y 方向突出）
%         140,   60,     60;       %  球体 18:  下方群右侧球
%         20,     100,   100;     %  球体 19:  零散小球（最左侧）
%         100,   20,     100;     %  球体 20:  零散小球（最下方）
%         140,   100,   100;     %  球体 21:  零散小球（最右侧）
%         100,   140,   100;     %  球体 22:  零散小球（最上方）
%         100,   100,   20;       %  球体 23:  零散小球（最前方，Z 最小）
%         100,   100,   140       %  球体 24:  零散小球（最后方，Z 最大）
% ];
%
% %  与上述球心对应的半径向量（24 行 1 列，顺序与 circleCenter 完全一致）
% r = [
%         30;       %  球体 1:  中心大球半径
%         25;       %  球体 2:  中心上方球半径
%         25;       %  球体 3:  中心右侧球半径
%         25;       %  球体 4:  中心前方球半径
%         20;       %  球体 5:  左侧群大球半径
%         15;       %  球体 6:  左侧群中间球半径
%         15;       %  球体 7:  左侧群上方球半径
%         15;       %  球体 8:  左侧群右侧球半径
%         20;       %  球体 9:  右侧群大球半径
%         15;       %  球体 10:  右侧群中间球半径
%         15;       %  球体 11:  右侧群下方球半径
%         15;       %  球体 12:  右侧群左侧球半径
%         15;       %  球体 13:  上方群左侧球半径
%         20;       %  球体 14:  上方群大球半径
%         15;       %  球体 15:  上方群右侧球半径
%         15;       %  球体 16:  下方群左侧球半径
%         20;       %  球体 17:  下方群大球半径
%         15;       %  球体 18:  下方群右侧球半径
%         10;       %  球体 19:  最左侧零散小球半径
%         10;       %  球体 20:  最下方零散小球半径
%         10;       %  球体 21:  最右侧零散小球半径
%         10;       %  球体 22:  最上方零散小球半径
%         10;       %  球体 23:  最前方零散小球半径
%         10         %  球体 24:  最后方零散小球半径
% ];
% circleCenter = [
%         100,   100,   100;     %  球体 1:  中心区域主球
%         130,     90,   110;     %  球体 2:  中心区域右前方
%         80,     120,     90;     %  球体 3:  中心区域左上方
%         110,     70,   120;     %  球体 4:  中心区域右下方
%         50,       50,     60;     %  球体 5:  左侧区域主球
```

```
%       30,    100,    80;    % 球体 6: 左侧区域中间（X 最小）
%       60,    140,   100;    % 球体 7: 左侧区域右上方
%       40,     80,   130;    % 球体 8: 左侧区域上方（Z 较大）
%       150,    60,    70;    % 球体 9: 右侧区域主球
%       170,   110,    90;    % 球体 10: 右侧区域中间（X 最大）
%       140,   150,   110;    % 球体 11: 右侧区域右上方
%       160,    80,   140;    % 球体 12: 右侧区域上方（Z 较大）
%       80,     60,   150;    % 球体 13: 上方区域左下方
%       120,   100,   160;    % 球体 14: 上方区域主球（Z 较大）
%       100,   140,   140;    % 球体 15: 上方区域上方（Y 较大）
%       90,     70,    50;    % 球体 16: 下方区域左上方
%       130,   120,    40;    % 球体 17: 下方区域主球（Z 最小）
%       70,    100,    60;    % 球体 18: 下方区域中间
%       20,    120,    90;    % 球体 19: 边缘最左侧（X 最小）
%       180,    80,   110;    % 球体 20: 边缘最右侧（X 最大）
%       100,    20,    70;    % 球体 21: 边缘最下方（Y 最小）
%       90,    180,   130;    % 球体 22: 边缘最上方（Y 最大）
%       60,     60,    20;    % 球体 23: 边缘最前方（Z 最小）
%       140,   140,   180;    % 球体 24: 边缘最后方（Z 最大）
%       25,    160,    50;    % 球体 25: 外围左上方（X 小 Y 大）
%       175,    40,   120;    % 球体 26: 外围右下方（X 大 Y 小）
%       50,    180,    90;    % 球体 27: 外围左上方（X 小 Y 最大）
%       150,    25,   160     % 球体 28: 外围右下方（X 大 Y 最小）
% ];
%
% % 与上述球心对应的半径向量（28 行 1 列，顺序与 circleCenter 完全一致）
% r = [
%       25;    % 球体 1: 中心主球半径
%       20;    % 球体 2: 中心右前方半径
%       20;    % 球体 3: 中心左上方半径
%       18;    % 球体 4: 中心右下方半径
%       18;    % 球体 5: 左侧主球半径
%       15;    % 球体 6: 左侧中间半径
%       15;    % 球体 7: 左侧右上方半径
%       12;    % 球体 8: 左侧上方半径
%       18;    % 球体 9: 右侧主球半径
%       15;    % 球体 10: 右侧中间半径
%       15;    % 球体 11: 右侧右上方半径
%       12;    % 球体 12: 右侧上方半径
%       15;    % 球体 13: 上方左下方半径
%       18;    % 球体 14: 上方主球半径
%       15;    % 球体 15: 上方上方半径
%       15;    % 球体 16: 下方左上方半径
%       18;    % 球体 17: 下方主球半径
```

```
%        15;     % 球体 18：下方中间半径
%        10;     % 球体 19：边缘最左侧半径
%        10;     % 球体 20：边缘最右侧半径
%        10;     % 球体 21：边缘最下方半径
%        10;     % 球体 22：边缘最上方半径
%        10;     % 球体 23：边缘最前方半径
%        10;     % 球体 24：边缘最后方半径
%        12;     % 球体 25：外围左上方半径
%        12;     % 球体 26：外围右下方半径
%        12;     % 球体 27：外围左上方半径
%        12      % 球体 28：外围右下方半径
% ];

    cylinderMatrix = [10,100;40,40];    % 圆柱体中心坐标
    cylinderRMatrix = [10;20];    % 圆柱体半径
    cylinderHMatrix = [200;150];    % 圆柱体的高

    % 绘图设置（显示过程）
    display = true;    % 显示中间过程
    fig = figure;
    [x, y, z] = sphere;
    for i = 1:length(circleCenter(:,1))
        mesh(r(i)*x + circleCenter(i,1), r(i)*y + circleCenter(i,2), r(i)*z + circleCenter(i,3));
        hold on;
    end
    axis equal;
    title('双向 RRT 路径规划过程');
    xlabel('X');
    ylabel('Y');
    zlabel('Z');

    %% 参数设置
    source = [10 10 10];    % 起点
    goal = [150 150 150];    % 终点
    stepsize = 10;
    threshold = 10;
    maxFailedAttempts = 10000;
    searchSize = [200 200 200];    % 探索空间六面体
    sampleCount = 0;    % 采样点数统计

    %% 绘制起点和终点
    hold on;
    scatter3(source(1), source(2), source(3), 50, "filled", "g");
    text(source(1) + 5, source(2) + 5, source(3) + 5, 'start', 'Color', 'g', 'FontWeight', 'bold');
```

```
    scatter3(goal(1), goal(2), goal(3), 50, "filled", "b");
    text(goal(1) + 5, goal(2) + 5, goal(3) + 5, 'final', 'Color', 'b', 'FontWeight', 'bold');

    tic;   % 计时开始
    RRTree1 = double([source -1]);   % 起点树
    RRTree2 = double([goal -1]);       % 终点树
    tree1ExpansionFail = false;
    tree2ExpansionFail = false;
    pathFound = [];

    %% 双向扩展
    while ~tree1ExpansionFail || ~tree2ExpansionFail
        % 扩展第一棵树（从起点）
        if ~tree1ExpansionFail
            sampleCount = sampleCount + 1;
            [RRTree1, pathFound, tree1ExpansionFail, closestNode, newPoint] = ...
                rrtExtend3(RRTree1, RRTree2, goal, stepsize, maxFailedAttempts, threshold, ...
                circleCenter, r, searchSize, cylinderMatrix, cylinderRMatrix, cylinderHMatrix);

            if display && ~tree1ExpansionFail && isempty(pathFound)
                randomColor = rand(1, 3);
                plot3([closestNode(1);     newPoint(1)],     [closestNode(2);     newPoint(2)],
[closestNode(3); newPoint(3)], ...
                    'LineWidth', 1, 'Color', randomColor);
                drawnow;
            end
        end

        % 扩展第二棵树（从终点）
        if ~tree2ExpansionFail
            sampleCount = sampleCount + 1;
            [RRTree2, pathFound, tree2ExpansionFail, closestNode, newPoint] = ...
                rrtExtend3(RRTree2,    RRTree1,    source,    stepsize,    maxFailedAttempts,
threshold, ...
                circleCenter, r, searchSize, cylinderMatrix, cylinderRMatrix, cylinderHMatrix);

            if ~isempty(pathFound)
                pathFound(4:5) = pathFound(5:-1:4);   % 交换索引
            end
            if display && ~tree2ExpansionFail && isempty(pathFound)
                randomColor = rand(1, 3);
                plot3([closestNode(1);     newPoint(1)],     [closestNode(2);     newPoint(2)],
[closestNode(3); newPoint(3)], ...
                    'LineWidth', 1, 'Color', randomColor);
```

```
            drawnow;
        end
    end

    % 检查是否找到路径
    if ~isempty(pathFound)
        % 生成完整路径
        path = [pathFound(1,1:3)];
        prev = pathFound(1,4);
        % 回溯第一棵树
        while prev > 0
            path = [RRTree1(prev,1:3); path];
            prev = RRTree1(prev,4);
        end
        % 回溯第二棵树
        prev = pathFound(1,5);
        while prev > 0
            path = [path; RRTree2(prev,1:3)];
            prev = RRTree2(prev,4);
        end
        break;
    end
end

if isempty(pathFound)
    error('未找到路径，已达到最大尝试次数');
end

% 计算路径长度
pathLength = 0;
for i = 1:length(path(:,1))-1
    pathLength = pathLength + distanceCost3(path(i,1:3), path(i+1,1:3));
end

% 计算总用时
totalTime = toc;

% 绘制障碍物和起点、终点
figure; % 新图形窗口
[x, y, z] = sphere;
for i = 1:length(circleCenter(:,1))
    mesh(r(i)*x + circleCenter(i,1), r(i)*y + circleCenter(i,2), r(i)*z + circleCenter(i,3));
    hold on;
end
```

```
    axis equal;
    title('双向 RRT 路径规划 - 最终路径');
    xlabel('X');
    ylabel('Y');
    zlabel('Z');
    hold on;
    scatter3(source(1), source(2), source(3), 50, "filled", "g");
    text(source(1) + 5, source(2) + 5, source(3) + 5, 'start', 'Color', 'g', 'FontWeight', 'bold');
    scatter3(goal(1), goal(2), goal(3), 50, "filled", "b");
    text(goal(1) + 5, goal(2) + 5, goal(3) + 5, 'final', 'Color', 'b', 'FontWeight', 'bold');
    % 绘制最终路径
    plot3(path(:,1), path(:,2), path(:,3), 'r-', 'LineWidth', 2);
end
```
