# Supplementary material for: Path planning for volumetric flask grasping based on visual guidance and multi-constraint optimization
Source: PLoS One. 2026 Apr 20;21(4):e0347043. doi: 10.1371/journal.pone.0347043 (PMC13095110; doi:10.1371/journal.pone.0347043)
Supplement: S1 File — This file contains MATLAB-related code and experimental data to reproduce the results presented in the manuscript. (ZIP) [file pone.0347043.s001.zip › 支持信息/RRTStar.pdf]

```
function [path, T, sampleCount] = RRTStar(axisStart,axisLWH,startPoint,goalPoint,cubeInfo,cylinderInfo,sphereInfo)
    % 初始化采样点数
    sampleCount = 0;
    iterMax = 10000;                    %最大迭代次数
    iter = 0;                           %当前迭代次数
    step = 10;                          %步长
    count = 1;                          %计数器
    Thr = 10;                           %阈值
    randProbability = 0.9;              %随机采样概率，范围 0-1 之间，越大随机性越大。越小导向性越大，收敛快
    r = 5*step;                         %影响范围，若大一点路径规划效果好，但是迭代慢。若小一点，路径规划效果和 rrt 算法越贴近
    flag = 0;                           %路径规划参数，当规划失败时返回 0，规划成功返回 1
    %%%%%%%%%%    配置树的信息    %%%%%%%%%%
    T.x(1) = startPoint(1);
    T.y(1) = startPoint(2);
    T.z(1) = startPoint(3);
    T.pre(1) = 0;
    T.cost(1) = 0;
    path = [];

    while iter <= iterMax
        % 迭代次数加 1
        iter = iter + 1;
        % 每次迭代采样次数加 1，这里简单示例，你可根据实际采样逻辑调整
        sampleCount = sampleCount + 1;
        % 空间中随机采样
        randCoor = RandSample(axisStart,axisLWH,goalPoint,randProbability);

        % 寻找树上最近的点
        [nearestCoor,parentIndex] = FindNearstPoint(T,randCoor);

        % 根据指定步长扩展新的点
        newCoor = ExpandPoint(nearestCoor,randCoor,step);

        % 重写
        parentIndex = RewriteFunction(T,newCoor,r,parentIndex);

        % 碰撞检测
        A = [T.x(parentIndex),T.y(parentIndex),T.z(parentIndex)];
        B = newCoor;
        collisionFlag =
```

```
CollisionDetection(cubeInfo,cylinderInfo,sphereInfo,A,B,CalcuDistance(A,B));

        if collisionFlag
            continue;
        end

        % 将新点插入进来
        count = count + 1;
        T.x(count) = newCoor(1);
        T.y(count) = newCoor(2);
        T.z(count) = newCoor(3);
        T.pre(count) = parentIndex;
        T.cost(count) = CalcuDistance(A,B)+T.cost(parentIndex);

        branchColor = rand(1,3);
        line([A(1),B(1)],[A(2),B(2)],[A(3),B(3)],...
        'Color', branchColor, ...
        'LineWidth', 0.8);
        pause(0.01)


        T = RandRelink(T,newCoor,cubeInfo,cylinderInfo,sphereInfo,step,r);

        if CalcuDistance(newCoor,goalPoint)<Thr
            flag = 1;
            break;
        end

    end

    % 路径规划失败直接返回
    if ~flag
        disp('路径规划失败');
        return;
    else
        disp('路径规划成功');
    end

    % 寻找路径
    path = FindPath(T,startPoint,goalPoint);
end
```
