# Supplementary material for: Path planning for volumetric flask grasping based on visual guidance and multi-constraint optimization
Source: PLoS One. 2026 Apr 20;21(4):e0347043. doi: 10.1371/journal.pone.0347043 (PMC13095110; doi:10.1371/journal.pone.0347043)
Supplement: S1 File — This file contains MATLAB-related code and experimental data to reproduce the results presented in the manuscript. (ZIP) [file pone.0347043.s001.zip › 支持信息/RRTStarWithVisualSampling.pdf]

```
function [path,T,randomSampleCount] = RRTStarWithVisualSampling_CountRandom(axisStart,axisLWH,startPoint,goalPoint,cubeInfo,cylinderInfo,sphereInfo)
    %% 变量定义
    iterMax = 10000;                    % 最大迭代次数
    iter = 0;                           % 当前迭代次数
    step = 10;                          % 步长
    count = 1;                          % 树节点计数器
    Thr = 10;                           % 到达目标阈值
    randProbability = 0.9;              % 随机采样概率
    r = 5*step;                         % 父节点搜索范围
    flag = 0;                           % 路径成功标志
    goalBias = 0.8;                     % 目标偏向采样概率
    randomSampleCount = 0;              % 采样点的计数器

    % 初始化树结构
    T.x(1) = startPoint(1);
    T.y(1) = startPoint(2);
    T.z(1) = startPoint(3);
    T.pre(1) = 0;
    T.cost(1) = 0;
    path = [];

    while iter <= iterMax
        iter = iter + 1;

        %% 视觉引导采样
        if rand() < goalBias
            randCoor = goalPoint + 0.2*step*(rand(1,3)-0.5);
        else
            randCoor = RandSample(axisStart,axisLWH,goalPoint,randProbability);
            randomSampleCount = randomSampleCount + 1;
        end

        %% 后续逻辑不变（寻找最近点、扩展新点等）
        [nearestCoor,parentIndex] = FindNearstPoint(T,randCoor);
        newCoor = ExpandPoint(nearestCoor,randCoor,step);
        parentIndex = RewriteFunction(T,newCoor,r,parentIndex);

        A = [T.x(parentIndex), T.y(parentIndex), T.z(parentIndex)];
        B = newCoor;
        collisionFlag = CollisionDetection(cubeInfo,cylinderInfo,sphereInfo,A,B,CalcuDistance(A,B));
```

```
        if collisionFlag
            continue;
        end

        count = count + 1;
        T.x(count) = newCoor(1);
        T.y(count) = newCoor(2);
        T.z(count) = newCoor(3);
        T.pre(count) = parentIndex;
        T.cost(count) = CalcuDistance(A,B) + T.cost(parentIndex);

        % 绘制树枝
        branchColor = rand(1,3);
        line([A(1),B(1)],[A(2),B(2)],[A(3),B(3)],...
             'Color', branchColor, ...
             'LineWidth', 0.8);
        pause(0.01);

        T = RandRelink(T,newCoor,cubeInfo,cylinderInfo,sphereInfo,step,r);

        if CalcuDistance(newCoor,goalPoint) < Thr
            flag = 1;
            break;
        end
    end

    %% 生成路径
    if ~flag
        disp('路径规划失败：未找到可行路径');
        path = [];
        return;
    else
        disp('路径规划成功：找到可行路径');
    end
    path = FindPath(T,startPoint,goalPoint);
end
```
