# Supplementary material for: Path planning for volumetric flask grasping based on visual guidance and multi-constraint optimization
Source: PLoS One. 2026 Apr 20;21(4):e0347043. doi: 10.1371/journal.pone.0347043 (PMC13095110; doi:10.1371/journal.pone.0347043)
Supplement: S1 File — This file contains MATLAB-related code and experimental data to reproduce the results presented in the manuscript. (ZIP) [file pone.0347043.s001.zip › 支持信息/samplePoint_improve.pdf]

```
function randCoor = samplePoint_improve(axisStart,axisLWH,goalPoint,startPoint,findpath,totalcost)
if findpath == 0
    if rand<0.5
        randX = rand*axisLWH(1)+axisStart(1);
        randY = rand*axisLWH(2)+axisStart(2);
        randZ = rand*axisLWH(3)+axisStart(3);
        randCoor = [randX randY randZ];

    else
        randCoor = goalPoint;
    end
else
    %赋初值，求 x,y,z 三轴的半轴长
    x1 = startPoint(1);
    y1 = startPoint(2);
    z1 = startPoint(3);
    x2 = goalPoint(1);
    y2 = goalPoint(2);
    z2 = goalPoint(3);
    dis = sqrt((x1-x2)^2+(y1-y2)^2+(z1-z2)^2);
    a = 0.5*totalcost;
    b = 0.5*sqrt(totalcost^2-dis^2);
    c = b;
    %随机在一个以 a 为半径的球体内生成一个点
    angle1=rand(1)*2*pi;
    angle2=acos(rand(1)*2-1);
    r=a*power(rand(1),1/3);
    x=r.*cos(angle1).*sin(angle2);
    y=r.*sin(angle1).*sin(angle2);
    z=r.*cos(angle2);

    %%将点映射到椭球体上
    x = x;
    y = b*y/a;
    z = c*z/a;
    %旋转和平移
    an1 =atan2(x2-x1,y2-y1);
    an2 =-atan2(z2-z1,sqrt((x1-x2)^2+(y1-y2)^2));

    Rz=[cos(an1) -sin(an1) 0;
        sin(an1) cos(an1) 0;
        0 0 1];
    Ry=[cos(an2) 0 sin(an2);
```

```
            0 1 0;
            -sin(an2) 0 cos(an2)];
%       Rx=[1 0 0;
%           0 cos(an1) -sin(an1);
%           0 sin(an1) cos(an1)];
    n=Ry*Rz*[x;y;z];
    xn = n(1);
    yn = n(2);
    zn = n(3);
%       k1 =(xn/a)^2+(yn/b)^2+(zn/c)^2;
    x = xn +(x1+x2)/2;
    y = yn +(y1+y2)/2;
    z = zn +(z1+z2)/2;
    if x < axisStart(1)
        x =axisStart(1);
    end
    if y < axisStart(2)
        y =axisStart(2);
    end
    if z < axisStart(3)
        z =axisStart(3);
    end
    if x > axisLWH(1)
        x = axisLWH(1);
    end
    if y > axisLWH(2)
        y = axisLWH(2);
    end
    if z > axisLWH(3)
        z = axisLWH(3);
    end
    randCoor =[x y z];

end
end
```
