# Supplementary material for: Path planning for volumetric flask grasping based on visual guidance and multi-constraint optimization
Source: PLoS One. 2026 Apr 20;21(4):e0347043. doi: 10.1371/journal.pone.0347043 (PMC13095110; doi:10.1371/journal.pone.0347043)
Supplement: S1 File — This file contains MATLAB-related code and experimental data to reproduce the results presented in the manuscript. (ZIP) [file pone.0347043.s001.zip › 支持信息/TOPPRA.pdf]

```
%% 机械臂轨迹规划 - 完整显示+Y/Z 轴
clear all; clc; close all;
startup_rvc;

%% 1. 基础参数与机器人建模
a2=600; a3=100; d2=200; d4=600;
L1=Link ('d',0,'a',0,'alpha',0,'modified');
L2=Link ('d',d2,'a',0,'alpha',-pi/2,'modified'); L2.offset=-pi/2;
L3=Link ('d',0,'a',a2,'alpha',0,'modified');
L4=Link ('d',d4,'a',a3,'alpha',-pi/2,'modified');
L5=Link ('d',0,'a',0,'alpha',pi/2,'modified');
L6=Link ('d',0,'a',0,'alpha',-pi/2,'modified');
PUMA=SerialLink ([L1 L2 L3 L4 L5 L6],'name','AUBOI5');

%% 2. RTT 路径生成
source=[800 0 0]; goal=[700,700,800];
RadiusForNeib = 200;
circleCenterSave = [900,400,600; 800,300,300; 1100,200,400; 800,700,300];
LEN = length(circleCenterSave); rSave = [180; 150; 150; 120];
xx=[]; h=[]; circleCenter=circleCenterSave (1:LEN,:); r=rSave (1:LEN,:);

while length (xx)==0
    xx=[]; h=[];
    if ~isempty (h)
        close (h);
    end
    h=figure (1);
    [xx,yy,zz]=RTTPath (source,goal,circleCenter,r,RadiusForNeib,1);
end
myRobPath {1}=[xx',yy',zz']; mypos_iter=myRobPath {1};
disp('RTT 无碰撞路径生成完成');

%% 3. 轨迹优化
dt = 0.01;
TOTAL_TIME = 2;
t_dense = linspace(0, TOTAL_TIME, round(TOTAL_TIME/dt));

% -------------------- 方法 2：简化 B 样条 --------------------
new_Path = interp1(1:size(mypos_iter,1), mypos_iter, linspace(1, size(mypos_iter,1), length(t_dense)), 'spline');
vel2_raw = diff(new_Path, 1, 1) / dt;
vel2_raw = [vel2_raw; vel2_raw(end,:)];
accel2_raw = diff(new_Path, 2, 1) / dt^2;
accel2_raw = [accel2_raw; accel2_raw(end,:); accel2_raw(end,:)];
```

```
smoothVel2 = vel2_raw;
smoothAccel2 = accel2_raw;

% -------------------- 方法 1：基础差分法 --------------------
path1 = mypos_iter;
path1 = interp1(1:size(path1,1), path1, linspace(1, size(path1,1), length(t_dense)), 'linear');
vel1 = diff(path1, 1, 1) / dt;
vel1 = [vel1; vel1(end,:)];
accel1 = diff(path1, 2, 1) / dt^2;
accel1 = [accel1; accel1(end,:); accel1(end,:)];
smoothVel1 = vel1;
smoothAccel1 = accel1;

% -------------------- 方法 3：TOPP-RA 优化 --------------------
vel_limits = [1000, 1000, 1000];
accel_limits = [5000, 5000, 5000];
path3 = new_Path;
[optimized_path, time_vector, vel3, accel3] = toppra_optimize(path3, vel_limits, accel_limits);
vel3 = align_data_length(vel3, length(t_dense));
accel3 = align_data_length(accel3, length(t_dense));
smoothVel3 = vel3;
smoothAccel3 = accel3;

% =================== 速度对比（X/Y/Z 轴） ===================
figure('Name','速度对比（全轴）');
% X 轴速度
subplot(3,1,1);
plot(t_dense, smoothVel1(:,1), 'r--', 'LineWidth',1.5); hold on;
plot(t_dense, smoothVel2(:,1), 'b-', 'LineWidth',1.5);
plot(t_dense, smoothVel3(:,1), 'k-.', 'LineWidth',1.5);
title('X-axis speed comparison');
xlabel('Time (s)'); ylabel('Speed (mm/s)');
legend('Method 1','Method 2','Method 3'); grid on;
xlim([0, TOTAL_TIME]); % 固定时间轴范围

% Y 轴速度
subplot(3,1,2);
plot(t_dense, smoothVel1(:,2), 'r--', 'LineWidth',1.5); hold on;
plot(t_dense, smoothVel2(:,2), 'b-', 'LineWidth',1.5);
plot(t_dense, smoothVel3(:,2), 'k-.', 'LineWidth',1.5);
title('Y-axis speed comparison');
xlabel('Time (s)'); ylabel('Speed (mm/s)');
legend('Method 1','Method 2','Method 3'); grid on;
xlim([0, TOTAL_TIME]);
```

```
% Z 轴速度
subplot(3,1,3);
plot(t_dense, smoothVel1(:,3), 'r--', 'LineWidth',1.5); hold on;
plot(t_dense, smoothVel2(:,3), 'b-', 'LineWidth',1.5);
plot(t_dense, smoothVel3(:,3), 'k-.', 'LineWidth',1.5);
title('Z-axis speed comparison');
xlabel('Time (s)'); ylabel('Speed (mm/s)');
legend('Method 1','Method 2','Method 3'); grid on;
xlim([0, TOTAL_TIME]);

% ==================== 加速度对比（X/Y/Z 轴） ====================
figure('Name','加速度对比（全轴）');
% X 轴加速度
subplot(3,1,1);
plot(t_dense, smoothAccel1(:,1), 'r--', 'LineWidth',1.5); hold on;
plot(t_dense, smoothAccel2(:,1), 'b-', 'LineWidth',1.5);
plot(t_dense, smoothAccel3(:,1), 'k-.', 'LineWidth',1.5);
title('X-axis acceleration comparison');
xlabel('Time (s)'); ylabel('Acceleration (mm/s²)');
legend('Method 1','Method 2','Method 3'); grid on;
xlim([0, TOTAL_TIME]);

% Y 轴加速度
subplot(3,1,2);
plot(t_dense, smoothAccel1(:,2), 'r--', 'LineWidth',1.5); hold on;
plot(t_dense, smoothAccel2(:,2), 'b-', 'LineWidth',1.5);
plot(t_dense, smoothAccel3(:,2), 'k-.', 'LineWidth',1.5);
title('Y-axis acceleration comparison');
xlabel('Time (s)'); ylabel('Acceleration (mm/s²)');
legend('Method 1','Method 2','Method 3'); grid on;
xlim([0, TOTAL_TIME]);

% Z 轴加速度
subplot(3,1,3);
plot(t_dense, smoothAccel1(:,3), 'r--', 'LineWidth',1.5); hold on;
plot(t_dense, smoothAccel2(:,3), 'b-', 'LineWidth',1.5);
plot(t_dense, smoothAccel3(:,3), 'k-.', 'LineWidth',1.5);
title('Z-axis acceleration comparison');
xlabel('Time (s)'); ylabel('Acceleration (mm/s²)');
legend('Method 1','Method 2','Method 3'); grid on;
xlim([0, TOTAL_TIME]);

%% 辅助函数
```

```
function data_out = align_data_length(data_in, target_len)
    current_len = size(data_in,1);
    data_out = data_in;
    if current_len < target_len
        pad_rows = target_len - current_len;
        pad_data = repmat(data_in(end,:), pad_rows, 1);
        data_out = [data_in; pad_data];
    elseif current_len > target_len
        data_out = data_in(1:target_len, :);
    end
    if isempty(data_out)
        data_out = zeros(target_len, size(data_in,2));
    end
end

function [optimized_path, time_vec, vel_profile, accel_profile] = toppra_optimize(path, vel_lim,
accel_lim)
    N = size(path, 1);
    if N < 2
        optimized_path = path; time_vec = 0; vel_profile = zeros(size(path)); accel_profile =
zeros(size(path)); return;
    end
    s = zeros(N,1);
    for i = 2:N; s(i) = s(i-1) + norm(path(i,:) - path(i-1,:)); end
    total_s = s(end);
    if total_s == 0
        optimized_path = path; time_vec = linspace(0,1,N)'; vel_profile = zeros(size(path));
accel_profile = zeros(size(path)); return;
    end
    s = s / total_s;
    tangent = zeros(N, 3);
    tangent(1,:) = (path(2,:)-path(1,:))/norm(path(2,:)-path(1,:));
    for i = 2:N-1; tangent(i,:) = (path(i+1,:)-path(i-1,:))/norm(path(i+1,:)-path(i-1,:)); end
    tangent(N,:) = (path(N,:)-path(N-1,:))/norm(path(N,:)-path(N-1,:));
    v_max_s = zeros(N,1); a_max_s = zeros(N,1);
    for i = 1:N
        t_abs = abs(tangent(i,:)); t_abs(t_abs<1e-6)=1e-6;
        v_max_s(i) = min(vel_lim./t_abs); a_max_s(i) = min(accel_lim./t_abs);
    end
    v_forward = zeros(N,1);
    for i = 2:N
        ds_i = s(i)-s(i-1);
        v_forward(i) = min(sqrt(v_forward(i-1)^2 + 2*a_max_s(i)*ds_i*total_s), v_max_s(i));
    end
```

```
    v_backward = zeros(N,1);
    for i = N-1:-1:1
        ds_i = s(i+1)-s(i);
        v_backward(i) = min(sqrt(v_backward(i+1)^2 + 2*a_max_s(i)*ds_i*total_s), v_max_s(i));
    end
    v_opt_s = min(v_forward, v_backward); v_opt_s(v_opt_s<0)=0;
    time_vec = zeros(N,1);
    for i = 2:N
        ds_i = (s(i)-s(i-1))*total_s;
        dt = 2*ds_i/(v_opt_s(i)+v_opt_s(i-1)+1e-6);
        time_vec(i) = time_vec(i-1)+dt;
    end
    vel_profile = tangent .* repmat(v_opt_s,1,3);
    accel_profile = zeros(N,3);
    for i = 2:N-1
        dt_i = time_vec(i+1)-time_vec(i-1);
        accel_profile(i,:) = (vel_profile(i+1,:)-vel_profile(i-1,:))/dt_i;
    end
    accel_profile(1,:)=0; accel_profile(N,:)=0; optimized_path = path;
end
```
